# Evaluating the Functional Importance of Conformer‐Dependent Atomic Partial Charge Assignment

**DOI:** 10.1002/jcc.70112

**Published:** 2025-05-14

**Authors:** Meghan Osato, Hannah M. Baumann, Jennifer Huang, Irfan Alibay, David L. Mobley

**Affiliations:** ^1^ Department of Pharmaceutical Sciences University of California, Irvine Irvine California USA; ^2^ Open Free Energy Open Molecular Software Foundation Davis California USA; ^3^ Department of Chemistry University of California, Irvine Irvine California USA

**Keywords:** conformation, force field, free energy calculation, partial charge

## Abstract

Physics‐based methods such as protein‐ligand binding free energy calculations have been increasingly adopted in early‐stage drug discovery to prioritize promising compounds for synthesis. However, the accuracy of these methods is highly dependent on the details of the calculation and choices made while preparing the ligands and protein ahead of running calculations. During ligand preparation, researchers typically assign partial atomic charges to each ligand atom using a specific ligand conformation for charge assignment, often the input conformer. While it is a well‐known problem that partial charge assignment is dependent on conformation, little investigation has explored the downstream effects of varied partial charge assignment on free energy estimates. Preliminary benchmarks from the Open Free Energy Project show that generating partial charges from different input conformers leads to variation of up to ±5.3 kcal/mol in calculated relative binding free energies due to variation in partial charges alone. In this study, we more systematically explore this issue, investigating it in smaller systems using absolute hydration free energy calculations to reduce the degrees of freedom and sources of statistical error as compared to larger protein‐ligand systems. We investigate how differences in partial charge generation (such as those caused by input conformer choice, partial charge engine, and hardware) may lead to differences in calculated absolute hydration free energy (AHFE) values. We demonstrate that supplying different input conformers to a partial charge engine can result in atomic partial charge discrepancies of up to 0.681 e, resulting in differences in calculated AHFE of 6.9±0.1 kcal/mol. We find that even relatively small variations in partial charge assignment can result in notable differences in calculated AHFE. Thus, care should be taken when assigning partial charges to ensure reproducibility and accuracy of any resulting free energy calculations. We expect that these effects will be magnified in pharmaceutically relevant binding free energy calculations with additional degrees of freedom, more highly directional interactions than in water, and potentially more statistical error.

## 1 Introduction

Determination of candidates to advance through the drug development pipeline consumes years of time and resources. Scientists must identify and filter through thousands of candidate compounds through vigorous validation experiments to eventually have one clinically approved drug. Physics‐based methods such as protein‐ligand binding free energy calculations have been increasingly adopted in early‐stage drug discovery to reduce the time and cost of the early stages of drug discovery by reducing the number of costly experiments [1, 2, 3]. Binding free energy calculations, such as relative binding free energy (RBFE) calculation methods, serve to prioritize related compounds through their binding affinity [4, 5, 6]. Relatedly, less computationally expensive hydration free energies are related to the solubility of a molecule, which is important for predicting bioavailability [7].

The accuracy of these methods is highly dependent on the methods used and the choices made while preparing the proteins and small molecules ahead of running calculations [8, 9]. Most commonly, free energy methods use classical all‐atom force fields [10, 11] which model interactions as a combination of valence terms (bond, angle, and torsional potentials), Lennard‐Jones interactions, and classical Coulombic interactions from atom‐centered partial atomic charges. During ligand preparation, computational chemists assign partial charges to each atom in a given small molecule [12]. These atomic partial charges aim to model a given molecule's electrostatic interactions with its environment, and are particularly crucial for strong polar and hydrogen bonding interactions often present in binding sites. Thus, partial charges have a major impact on downstream calculated values such as free energy estimates [13, 14]. The nature of electrons and their densities makes them difficult to model, especially as static floating‐point values. While polarizable force fields are available and go beyond fixed atom‐centered partial charges, the classical fixed‐charge model is still the most common for free energy calculations. In fixed‐charge models, atomic partial charges are modeled by static floating point values [15, 16]. It is important to note, however, that partial charges are not a physical observable [17], meaning that no single set of partial charges is “best”. Rather, researchers typically assign static partial charges that attempt to replicate the electrostatic field or potential around the molecule that would be derived from suitable quantum chemical calculations. Overall, in the classical fixed‐charge models employed here, there is actually a relatively tight interplay between the choice of Lennard‐Jones parameters and the set of partial charges used, since the total interaction energy—as observed in a high‐level quantum chemical calculation—is typically approximated in the force field by the sum of a Lennard‐Jones and a Coulombic term. We do not investigate this issue here, but it is important to remember that the charge model is one significant factor impacting how well the resulting force field reproduces quantum chemical energy surfaces.

Here, we refer to the partial charges assigned to all atoms in a molecule based on a single input conformation as a partial charge set.

**FIGURE 1:**
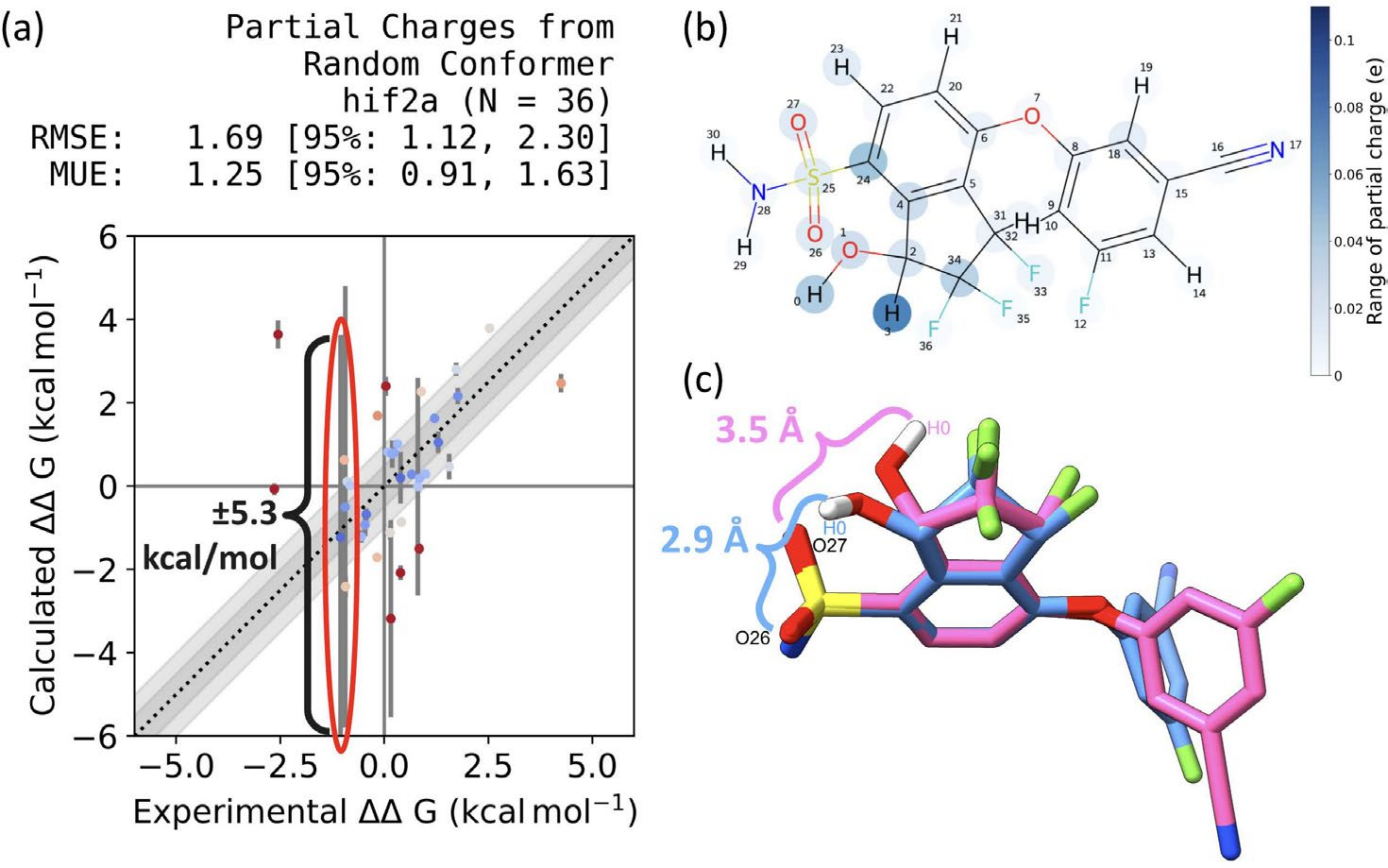
Variability in partial charges is associated with variability in calculated RBFE. (a) The data point for the molecule circled in red has a standard error across repeats of ±5.3 kcal/mol for its average calculated ΔΔG value. (b) The molecule corresponding to the circled data point in (a) is depicted in 2D. Atom H3 ranges in partial charge by 0.074 e. (c) The two input conformers that produce the most different partial charges at H3 are depicted. The blue conformer has O26 close in distance to H0, which may form a strong intramolecular interaction. The grey conformer has no atoms that form strong interactions with H0.

Not only are partial charge assignments method‐dependent, but some partial charge assignment methods also give charges that depend on the input conformer as well as the hardware on which the method is run. Partial charge methods which rely on quantum chemical calculations (including the AM1‐BCC method tested here) are sensitive to the input conformer because such methods rely on the molecular geometry (potentially after optimization) to estimate the electrostatics of the molecule [13, 18, 19, 20]. Conformers with strong intramolecular interactions or other geometric factors that impact the electronic structure can result in significantly different partial charges. As an example of a strong intramolecular interaction, consider an interaction between an oxygen and a hydrogen that are quite close to one another and not covalently bonded, in a scenario where the oxygen bears a negative partial charge and the hydrogen bears a positive partial charge. Following charge assignment, such a conformer will often result in different partial charges for the molecule than in conformers where the oxygen and hydrogen are rotated away from each other and not significantly interacting (Figure 1b,c). Different implementations of AM1‐BCC, such as those in AmberTools and the OpenEye toolkits, use different geometry optimization procedures and restraint choices, resulting in different “final” versions of the input conformers being used when the partial charge assignment finally takes place. While the partial charge algorithms themselves should, in theory, be deterministic and not depend on hardware, often implementations of these partial charge assignment methods also involve numerical analysis, which could be impacted by floating‐point precision and exact choice of computational algorithms [21]. For example, Mulliken population analysis, used in some charge assignment methods, including AM1 and related approaches, should be deterministic, but it is known to converge relatively poorly [22, 23]). Thus, compute hardware choice could potentially impact the resulting partial charge values, as we further examine in this work. Conformer dependence is probably the most substantial of these sources of variation, so some partial charge assignment methods attempt to remedy or reduce the conformer‐dependent effects. OpenEye's AM1‐BCC Electrostatically Least Interacting Functional Groups (ELF10) method filters input conformers to select only the top 10 conformers that have the fewest intramolecular interactions, and then averages the AM1‐BCC partial charges of the selected 10 conformers to produce AM1‐BCC ELF10 charges. Another method, Open Force Field's Not Another Graph Library (NAGL), is conformer‐independent (based only on the molecular graph) and produces the same charges irrespective of the input conformer.

While it is well known that partial charge assignment can vary significantly depending on charging method, little investigation has been done to describe the downstream effects of varied small molecule partial charge assignment on free energy estimates. In this study, we investigate the effects of differential partial charge assignment procedures on absolute hydration free energies (AHFEs). Past studies that investigated charge variation using hydration free energies typically investigated different sources of variation in partial charges than those studied here. For example, some studies examined random variations in partial charges or variations from changing the quantum chemical method for assigning charges. Several studies have investigated the downstream effect of assigned partial charges on AHFEs on a limited set of small molecules [24, 25]. One study found that different partial charge sets of a sucrose molecule resulted in maximal errors in the calculated AHFEs of 3.1 kcal/mol [26]. Another study found that a relative hydration free energy calculation between ethanol and propanol differed by up to 2.2 kcal/mol depending on the partial charge set used [27]. Lastly, two studies compared Restrained Electrostatic Potential (RESP) with HF/6‐31G* charges versus AM1‐BCC charge models, which were developed to behave similarly to RESP HF/6‐31G* charges. The resulting effects of each charge model on free energy calculations were inconclusive [28, 29, 30].

To an extent, it is hoped that these variations in partial charge sets have little functional importance for downstream applications such as free energy calculations. However, preliminary benchmarks from the Open Free Energy Project have shown that generating partial charges from different input conformers leads to high variability in calculated relative binding free energies of up to 9 kcal/mol (Figure 1a) [31, 32]. Different partial charges used in different studies of the same molecule will lead to different “correct” answers for calculated free energy values. This can adversely affect the rankings of compounds based on their calculated affinities and thus our recommendations we make to experimental researchers and pharmaceutical companies of compounds to prioritize in synthesis.

In this study, we investigate how much the partial charge engine makes a difference in partial charge variability across conformers for 126 molecules. We define conformer‐dependent partial charge variability as the partial charge differences that result from inputting different conformers of the same molecule into a partial charge engine and assigning partial charges. As we will investigate further in this work, this type of partial charge variability is affected by both the input conformers, the partial charge engine, and the hardware used for the calculation. Here, our focus is on AM1‐BCC charges as these are heavily used by the Open Force Field and Open Free Energy projects, which form the basis for our own typical binding free energy calculations. Particularly, we compare several different approaches for assigning AM1‐BCC charges—using AmberTools AM1‐BCC charges, OpenEye AM1‐BCC charges, and OpenEye AM1‐BCC ELF10 charges [13, 33, 34, 35]. We show that partial charge assignment can dramatically impact absolute hydration free energy calculations using 27 chemically diverse molecules (including binding free energies) due to apparently small variations in user input or even choice of computer architecture, meaning that significantly more effort may need to be paid by method developers to provide conformer‐independent charges.

## 2 Methods

### 2.1 Selection of Protein‐Ligand Benchmark Molecule Sets

We selected 5 molecule sets, where each molecule set contains ligands from one of five different targets from the Open Force Field Project's ProteinLigandBenchmark (PLB) repository, to use in our study [31, 32]. Four of the five molecule sets—HIF2a, P38a, PTP1b, SHP2—were chosen based on poor performance in previous benchmarks performed by the Open Free Energy Project, suspected to be caused by differences in partial charge assignment across repeats [36, 37]. The final molecule set, tyk2, was chosen due to the fact that it is normally well‐performing with small variation across identical repeats. Open Free Energy's benchmarks of these systems resulted in maximal variations of 0.38 kcal/mol across repeats for each molecule in the tyk2 set. These combined systems resulted in a set of 126 molecules.

Here, we focused on examining the *ligands* from these sets alone in solution, rather than in the binding site context, so we can focus our analysis on partial charge variation and its impact on molecular properties, rather than more demanding binding free energy calculations.

### 2.2 Truncation of Molecules

Because partial charge generation does not scale well with increasing numbers of atoms in a molecule, we sought to reduce the computational cost of this study by truncating uninteresting functional groups (and in particular, functional groups unlikely to impact the electrostatics of the remaining scaffold) from our molecules to reduce molecular size and the number of possible conformations, at least if truncated molecules still maintained the same level of partial charge variation (Figure S3). While the whole molecules may have greater conformer‐dependent charge variability, we still observed large variabilities in charge, even after truncating a subset of the molecules. We chose to truncate the HIF2a molecules for the remainder of the manuscript to reduce the computational cost of assigning charges, as AM1‐BCC charge assignment methods scale with the number of atoms in the molecule. We defined uninteresting functional groups as those that should have little conformational variation when charged, such as phenyl groups or symmetric aromatic rings. Because the HIF2a ligands were particularly large and flexible, which led to slow conformer generation and time‐consuming charge assignments, we sought to truncate these molecules (while checking to ensure that the same issues persisted) to identify more minimal versions that could be treated more rapidly while preserving the same issues. Each of the molecules was visually inspected for a functional group that could easily be truncated across a single bond based on our criteria. We only truncated the HIF2a molecules. Using an RDKit v2024.03.2 editableMolecule, we removed the functional group at the specified bond and added hydrogens to complete the valence of the atoms on the remaining molecule [38]. The truncated HIF2a molecules were used in place of the original HIF2a molecules for the remainder of our study (Figures S1 and S2).

### 2.3 Validation That Truncated Molecules Induce Partial Charge Variation

We validated that the truncated HIF2a molecules still induced partial charge variation by comparing the partial charge variation of the whole molecules to partial charge variation of the truncated molecules. We chose 5 HIF2a molecules at random and charged the whole and the truncated versions of the molecules. We confirmed that a subset of these molecules still produced conformer‐dependent partial charge variability. The truncated molecules still had large partial charge variations of over 0.09 e at a single atom. This level of variation is nearly identical to the level of variation seen in non‐truncated molecules. We also confirmed for a reference molecule that the truncated and whole molecule also produced comparable variability in calculated AHFE across 5 repeats of each (Figure S3).

### 2.4 Generation of Partial Charges

We generated partial charges for our 126 molecules using AmberTools AM1‐BCC charges, OpenEye AM1‐BCC charges, OpenEye AM1‐BCC ELF10 charges, and OpenFF NAGL charges [34, 39, 40].

#### 2.4.1 Single Conformer Partial Charge Generation Methods

Single conformer partial charge generation methods use one conformer as an input for atomic partial charge generation. We expected the charges generated from single conformer methods to differ depending on the choice of input conformer. As single conformer partial charge generation methods, we used AmberTools AM1‐BCC charges and OpenEye AM1‐BCC charges. Both of these methods perform a geometry optimization of the input conformer ahead of charging the molecules, though in the OpenEye case, this is a restrained geometry optimization, and the exact details of the optimization algorithm are not publicly available. We used the RDKit v2024.03.2 conformer generator starting from a random seed to generate 50 conformers for each molecule [38]. We charged each molecule 50 times, one for each conformer, with each partial charge generation method using openff‐toolkit v0.14.3, AmberTools v23.6, openeye‐toolkits v2023.2.3, and stored the partial charge sets for each conformer for later analysis [34, 39, 41]. For AmberTools AM1‐BCC charges, we used the default SQM settings for geometry optimization unless otherwise stated.

#### 2.4.2 Multi‐Conformer Partial Charge Generation Method

Multi‐conformer partial charge generation methods take multiple conformations as input and attempt to output a single “best” set of partial charges, thereby reducing conformer dependence. OpenEye's Electrostatically Least‐interacting Functional groups (ELF10) method works by averaging the partial charges of 10 diverse conformers from the top 2% of a molecule's conformers with the most favorable electrostatic intra‐molecular energies after all partial charges are made positive (this approach is designed to exclude conformers with strong internal electrostatic interactions). Here, using this approach (OpenEye AM1‐BCC ELF10), we generated input conformers using RDKit v2024.03.2. Conformers were generated starting from 50 different random seeds to generate 50 sets of 500 random conformers, the minimum required for OpenEye AM1‐BCC ELF10 charges [38]. We then charged each molecule 50 times, one for each set of 500 conformers, with the OpenEye AM1‐BCC ELF10 partial charge generation method using openff‐toolkit v0.14.3 and openeye‐toolkits v2023.2.3 [41].

#### 2.4.3 Conformer‐Independent Partial Charge Generation Method

Conformer‐independent charging methods are designed to generate partial charges that do not depend on the input conformer, for example, such as without using a quantum chemical method, by producing charges that depend only on the molecular graph. Here, for the conformer‐independent partial charge generation method, OpenFF NAGL, we used the 50 conformers generated for the AmberTools AM1‐BCC charges and OpenEye AM1‐BCC charges. The expectation was that generated charges would not depend on input conformer, since the method was designed to use only the molecular graph, but we varied input conformers to check this. We charged each conformer using openff‐toolkit v0.16.0 and openff‐nagl v0.3.8 [40, 42].

### 2.5 Metrics to Assess Partial Charge Variability

For our work, we used 2 metrics to assess the conformer‐dependent variation in partial charges, the maximal partial charge difference and the maximal Δq_bond difference.

#### 2.5.1 Maximal Partial Charge Difference Metric

The maximal partial charge difference measures the maximum variation in partial charge we observe across conformers for the same molecule with the same method. Using the partial charge generation methods relevant to this study, each atom in a molecule is assigned a floating‐point value as its partial charge. We will refer to the set of partial charges for all atoms in a molecule as a partial charge set. Within this work, partial charge sets may vary due to different input conformers, different hardware, and different partial charge generation methods.

To obtain the maximal partial charge difference for a molecule, we calculated the range of partial charges generated for each atom across all partial charge sets. We then took the maximal range seen for that molecule as the maximal partial charge difference. In other words, let A represent all the atoms in a molecule. Each atom, u, in the molecule will have a partial charge q(u) given a partial charge set, q. We define the maximal difference in partial charges across 2 sets of partial charges, $q_{1}$ and $q_{1}$, as:

(1) $$max(|q_{1}(u)-q_{2}(u)|,\forall (u)\in A)$$

#### 2.5.2 Maximal Δq_bond Difference Metric

The maximal Δq_bond difference looks at the difference in charge across each bond in a molecule, then finds the largest variation in charges across a bond induced by any assigned charge set.

We use the term “bond Δq”, represented in symbols as Δq_bond, to refer to the charge difference between two bonded atoms, or the charge seen across a bond, in an attempt to qualitatively describe the polarity of the bond. We refer to Δq_bond difference as the absolute value of the difference in Δq_bond across different partial charge sets. In other words, let B represent the set of all bonds in the molecule. Each atom, u, in the molecule will have a partial charge q(u). Each bond in B will be bound by 2 atoms, u and v. We define the set of all Δq_bond, QB, for a set of partial charges of a molecule as:

(2) QB={q(u)−q(v),∀(u,v)∈B}

Each set of partial charges will have a different set of Δq_bonds. We define the range of Δq_bond difference across 2 sets of partial charges $q_{1}$ and $q_{2}$ for a molecule as:

(3) $$max(|q_{1}(u)-q_{1}(v)-q_{2}(u)-q_{2}(v)|,\forall (u,v)\in B)$$

We define the maximal range of Δq_bond difference as the largest range of Δq_bond difference values seen at a bond of a molecule across all 50 partial charge sets. We use the metrics described above to compare the conformer‐dependent charge variability of each partial charge generation method of interest.

### 2.6 Selection of Molecules for AHFE Simulations

We selected molecules from the Protein‐Ligand Benchmarks (PLB) and FreeSolv databases for AHFE simulation.

#### 2.6.1 Selection of PLB Molecules for AHFE Simulations

In addition to looking at how much partial charges vary, we wanted to examine how these impact downstream calculations like free energy calculations. We selected the molecules for AHFE simulations by selecting 5 “good” molecules that produced low partial charge variation across 50 conformers, 5 “average” molecules that produced average partial charge variation across 50 conformers, and 5 “bad” molecules that produced high partial charge variation across 50 conformers. For all 126 molecules, we plotted the maximal partial charge differences for each molecule as a histogram. We used the OpenEye AM1‐BCC charges differences distribution to select our molecules for simulation, as this distribution was the most Gaussian. We selected 5 “good” molecules from the lower end of the distribution in the range of $\left[\bar{x}-1.8*\sigma ,\bar{x}-1*\sigma \right]$, 5 “average” molecules from the center of the distribution in the range of $\left[\bar{x}-0.4*\sigma ,\bar{x}+0.4*\sigma \right]$, and 5 “bad” molecules from the upper end of the distribution in the range of $\left[\bar{x}+1*\sigma ,\bar{x}+1.8*\sigma \right]$. These 15 molecules were used for the AHFE simulations.

#### 2.6.2 Selection of FreeSolv Molecules for AHFE Simulations

To compare calculated absolute hydration free energies to experimental data, we also ran AHFE calculations on a subset of the Mobley Lab FreeSolv database molecules [43]. Unlike molecules from the Protein‐Ligand Benchmarks set, these molecules have generally *not* been previously demonstrated to have partial charge variations that impact computed properties like binding free energies meaningfully; rather, these molecules are simply present in this widely used test set and are tested here to determine whether they are subject to conformational variation. We chose to examine them here in part to allow comparison to experimentally measured properties, and to see to what extent similar partial charge variation issues appear in this set. Thus, for each of the 642 molecules in the database, we generated 50 conformers using RDKit v2024.03.2 conformer generator starting from a random seed [38]. Each conformer was charged with AmberTools AM1‐BCC charges using openff‐toolkit v0.14.3 and AmberTools v23.6, resulting in 50 sets of partial charges for each molecule. We calculated the partial charge variation using the maximal Δq_bond difference across all partial charge sets for each molecule and ordered the molecules based from highest to lowest maximal Δq_bond difference. We changed the criteria after the analysis of the PLB compounds because we found that the maximal Δq_bond had a better correlation with the variability of the ΔG values than did the partial charge difference. We manually chose 12 molecules of the top 25 molecules with the highest maximal Δq_bond difference by avoiding molecules that had high similarity to those already chosen. We proceeded to run AHFE simulations on the 12 selected molecules using the preparation detailed below.

### 2.7 Molecule Preparation for AHFE Simulation

Each set of molecules—the PLB AHFE molecules and FreeSolv AHFE molecules—was prepared for AHFE simulations using the same protocol as described below. All molecule partial charges were generated on an M1 Mac unless otherwise stated.

#### 2.7.1 Preparation of PLB Simulations With Ambertools AM1‐BCC Charges

We generated 5 new random conformers for each molecule using RDKit v2024.03.2 conformer generator, different from those in Section 2.4 [38]. Each conformer was charged with AmberTools AM1‐BCC charge generation using openff‐toolkit v0.14.3 and AmberTools v23.6, resulting in 5 sets of partial charges for each molecule. For the PLB AHFE molecules, the conformer provided in the PLB molecule structural data file (SDF) was used as the starting conformer for the AHFE simulation. For the FreeSolv molecules, we generated a starting conformer using openeye‐toolkits v2023.2.3 oeomega. For each molecule, we generated 5 copies of the molecule with 3D coordinates matching the selected starting conformer. Each copy of the molecule was assigned one of the 5 partial charge sets previously generated, resulting in 5 nearly identical repeats of each molecule, only differing in their assigned partial charges. Each molecule was parametrized using openff‐2.0.0 small molecule force field [44]. Each molecule and its conformers were saved with the OpenEye oeb.gz format.

#### 2.7.2 Preparation of PLB Simulations With OpenEye AM1‐BCC ELF10 Charges

We selected 6 out of the 15 PLB molecules from the AmberTools AM1‐BCC simulations to study further. For each category of molecule, “good”, “average”, and “bad”, we selected the 2 molecules with the most variable calculated ΔGs. Using the conformers from Section 2.7.1, we charged each conformer using the AM1‐BCC ELF10 charge engine from openeye‐toolkits v2023.2.3 oequacpac. For each molecule, we generated 5 copies of the molecule with 3D coordinates matching the selected starting conformer. Each copy of the molecule was assigned one of the 5 partial charge sets previously generated resulting in 5 nearly identical repeats of each molecule only differing in their assigned partial charges (this was done to ensure that the *only* difference in our calculations was the assigned partial charges, not the starting conformer). Each molecule was parametrized using the openff‐2.0.0 small molecule force field.

#### 2.7.3 Preparation of PLB Simulations With OpenFF NAGL Charges

Using the same 6 molecules out of the 15 PLB molecules Section 2.7.2 and the same conformers from Section 2.7.1, we charged each conformer using the OpenFF NAGL charge engine v0.3.8 from openff‐toolkit v0.16.3 [40]. For each molecule, we generated 5 copies of the molecule with 3D coordinates matching the selected starting conformer. Each copy of the molecule was assigned one of the 5 partial charge sets previously generated, resulting in 5 nearly identical repeats of each molecule, only differing in their assigned partial charges. Each molecule was parametrized using the openff‐2.0.0 small molecule force field.

#### 2.7.4 Preparation of PLB Simulations With Ambertools AM1‐BCC Charges and Varying Hardware

Using the same 6 molecules out of the 15 PLB molecules Section 2.7.2 and the same conformers from Section 2.7.1, we charged each conformer using the AmberTools AM1‐BCC charge engine on an Intel processor. For each molecule, we generated 5 copies of the molecule with 3D coordinates matching the selected starting conformer. Each copy of the molecule was assigned one of the 5 partial charge sets previously generated, resulting in 5 nearly identical repeats of each molecule, only differing in their assigned partial charges. Each molecule was parametrized using the openff‐2.0.0 small molecule force field. We ensured that the linear algebra libraries on both the Intel and M1 Mac processors were equivalent (libblas v3.9.0 and liblapac v3.9.0 for both processors) to reduce confounding variables.

#### 2.7.5 Preparation of FreeSolv Simulations Using Ambertools AM1‐BCC

We generated 5 new random conformers for each molecule using the RDKit v2024.03.2 conformer generator, different from those in Section 2.4. Each conformer was charged with AmberTools AM1‐BCC charge generation using openff‐toolkit v0.14.3 and AmberTools v23.6, resulting in 5 sets of partial charges for each molecule. We generated a starting conformer using openeye‐toolkits v2023.2.3 oeomega. For each molecule, we generated 5 copies of the molecule with 3D coordinates matching the selected starting conformer. Each copy of the molecule was assigned one of the 5 partial charge sets previously generated, resulting in 5 nearly identical repeats of each molecule, only differing in their assigned partial charges. Each molecule was parametrized using the openff‐2.0.0 small molecule force field.

### 2.8 AHFE Simulation Details

For each absolute hydration free energy calculation, we ran 5 repeats of each molecule, where the only difference among the repeats was the partial charges. Molecule starting conformations and 3D coordinates of the molecule were held constant across each repeat. All AHFE simulations were run using Intel processors. All absolute hydration free energy simulations were run using the openfe v0.15.0 absolute solvation free energy protocol using water as the solvent [28, 45, 46]. The OpenFE solvation free energy protocol applies a Langevin middle integrator for Langevin dynamics and uses a Monte Carlo barostat. The calculation itself is split into two legs, annihilation in solvent and annihilation in vacuum. In all legs of the simulation, Coulomb interactions were turned off over 7 linearly spaced lambda windows, followed by turning off Van der Waals interactions over 13 linearly spaced lambda windows. Vacuum simulation legs were run for 0.5 ns equilibration and 1 ns production. Solvent simulations were run for 0.5 ns equilibration and 10 ns production. The online analysis target error setting, which terminates the simulation early if convergence is detected, was set to 0 to allow simulations to run to their full length. All other simulation settings were left at their default values. Scripts to set up simulations, as well as the input .json files, can be found on the GitHub repository associated with this paper. We confirmed that each simulation repeat had converged by plotting the calculated ΔG versus time and visually inspecting that each repeat had plateaued. Free energy estimates are obtained using a multistate Bennett acceptance ratio (MBAR) [47] estimator from PyMBAR [48].

### 2.9 Comparison of Partial Charges Generated From Differing Hardware

To compare the effect of hardware on partial charge generation, we charged the same conformers using AmberTools AM1‐BCC charges on Intel and M1 Mac processors. We generated 50 conformers for each of the 6 molecules from the 15 PLB molecules (Section 2.6.1) using RDKit v2024.03.2 conformer generator [38] on an M1 Mac processor. These conformers were saved within multi‐conformer molecules in the oeb.gz format. We loaded in the multi‐conformer molecule oeb.gz files and charged each of the 50 stored conformers for each molecule using AmberTools AM1‐BCC partial charges on an M1 Mac processor. We then replicated this process and loaded in the multi‐conformer molecule oeb.gz files, then charged the same 50 conformers for each molecule using AmberTools AM1‐BCC partial charges on an Intel processor. We compared the set of partial charges generated for each conformer on the M1 Mac processor versus the Intel processor using the metrics in Section 2.5.

## 3 Results

In this study, we investigate the extent to which different conformers and hardware can influence the partial charges generated for a molecule. We then test whether the variability in partial charges is functionally important in downstream free energy calculations—here, especially in hydration free energy calculations. Finally, we investigate the relationship between the magnitude of partial charge variability and the magnitude of discrepancies in calculated free energies.

### 3.1 Assigned Partial Charges Differ With Choice of Partial Charge Generation Method

We compare different partial charge assignment methods across our 5 sets of Protein‐Ligand Benchmark (PLB) molecules (Section 2.1) and find that slightly different charging methods can result in substantially different partial charges for a molecule. For clarity, we will refer to the unique set of partial charges assigned to each atom in a molecule as a partial charge set.

**FIGURE 2:**
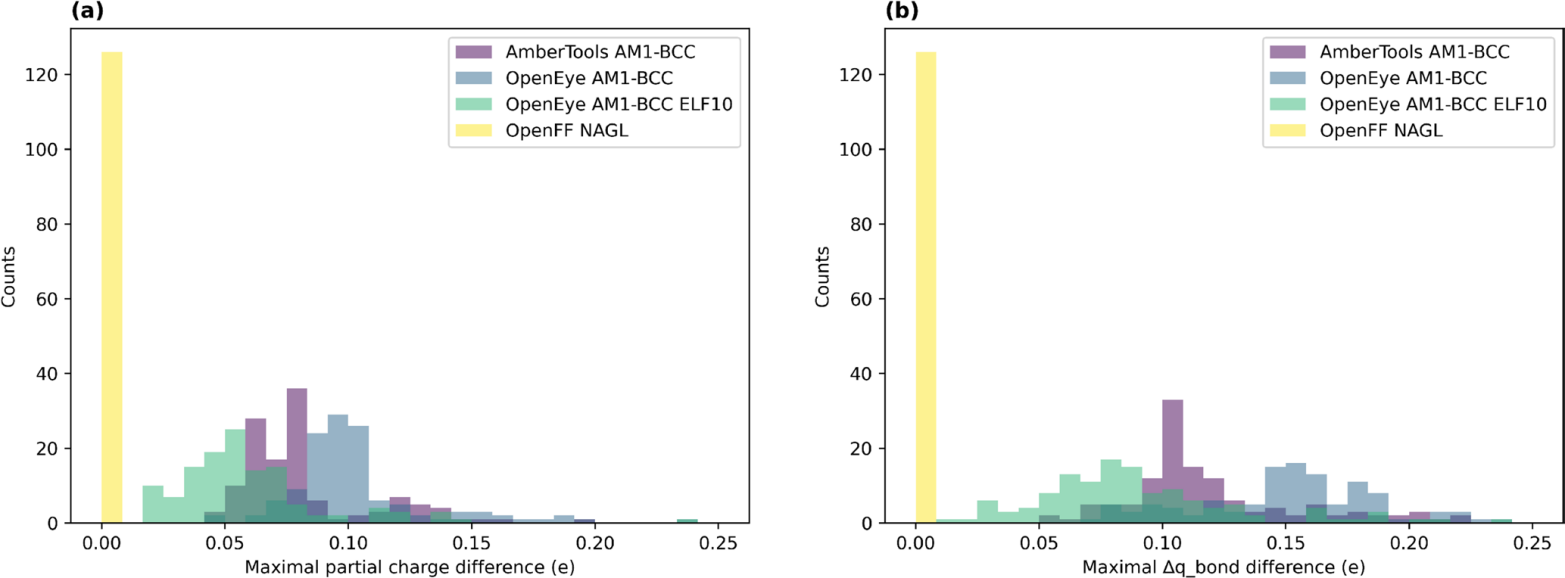
Differences between partial charge generation methods. Different charge assignment protocols/implementations result in different levels of conformer dependence for assigned partial charges, depending on the input conformer(s) used for partial charge generation. We generated 50 partial charge sets of each molecule using 4 different partial charge methods: Antechamber AM1‐BCC, OpenEye AM1‐BCC, OpenEye AM1‐BCC ELF10, and OpenFF NAGL. (a) Using maximal partial charge difference as our metric, Antechamber AM1‐BCC charges on average showed less variation than the OpenEye equivalent AM1‐BCC charges, and OpenEye AM1‐BCC ELF10 charges showed slightly less variation on average. As expected, OpenFF NAGL showed no conformer dependence. (b) Using our second metric, maximal Δq_bond difference, OpenFF NAGL again showed no conformer dependence. Again, on average, OpenEye AM1‐BCC ELF10 charges showed less variation on average as compared to Antechamber and OpenEye AM1‐BCC charges.

In this study, we use AmberTools AM1‐BCC, OpenEye AM1‐BCC, OpenEye AM1‐BCC ELF10, and Open Force Field's NAGL partial charge generation methods (Figure 2). We then evaluate the conformer dependence of a method by looking at the variability in partial charge that each method produces across 50 conformers or 50 conformer sets for a molecule using two different metrics maximal range of partial charge variability and maximal range of Δq_bond difference.

We find that single‐conformer partial charge generation methods, specifically AmberTools AM1‐BCC and single‐conformer OpenEye AM1‐BCC, exhibit more partial charge variability (as judged by either metric) on average than OpenEye AM1‐BCC ELF10 charges. The AM1‐BCC ELF10 charge is designed to be relatively conformer‐independent, but it requires at least 10 conformers and recommends a set of at least 500 conformers as input. For our AM1‐BCC ELF10 charge generation, we use the recommended 500 conformers. (Figure 2). Again, using both metrics, single‐conformer OpenEye AM1‐BCC is the most variable method, followed by AmberTools AM1‐BCC and OpenEye AM1‐BCC ELF10. OpenFF's NAGL has 0 variability across all molecules using both metrics as expected, since NAGL is a conformer‐independent method, and thus produces the same partial charges irrespective of the input conformer.

Overall, we find that different partial charge assignment methods can result in significantly different partial charges depending on the choice of input conformer, with charges varying in some cases by up to 0.23 e (Figure 2a). We will explore below (Section 3.3) whether this level of variation is functionally important for free energy calculations.

### 3.2 Assigned Partial Charges Differ With Choice of Hardware

Important numerical aspects of partial charge assignment could potentially be impacted by the computing environment used for the calculations, so we examine the impact of compute hardware on partial charge variation. We assess the effect of hardware on individual partial charge generation by assigning charges using an M1 Mac and cross‐comparing with results on an Intel processor. For each of our 6 molecules (Section 2.6.1), we generated and stored 50 conformers of each molecule. We charged each conformer on an Intel processor and again on an M1 Mac processor. Thus, for any given molecule, we obtain two sets of partial charges generated from the exact same conformers. Given two partial charge sets generated from conformer1, partialcharges1_mac generated on an M1 Mac, and partialcharges1_intel generated on an Intel processor, we find the maximal difference in partial charge seen at an atom. If hardware has no effect on partial charge generation, we expect each of these methods to produce the exact same or very similar partial charge sets, given the same input conformer and the same charge assignment method.

**FIGURE 3:**
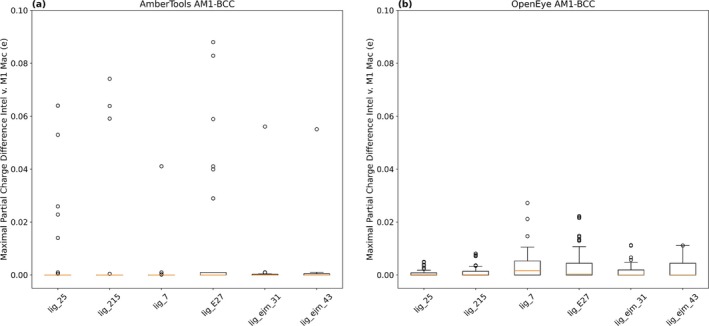
Hardware differences effects on partial charge generation. Differences in hardware result in different amounts of variation in assigned partial charges given the same input conformer. Each box and whisker plot represents the maximal partial charge difference seen between the same starting conformer charged on an Intel processor and an M1 Mac using the specified charging method—AmberTools AM1‐BCC or OpenEye AM1‐BCC. (a) On average, using AmberTools AM1‐BCC charges, the maximal absolute value of the partial charge difference between the same starting conformer charged on the Intel processor and an M1 Mac was nearly 0, but there were instances of differences up to 0.09 e. (b) On average, using OpenEye AM1‐BCC charges, the absolute maximal partial charge difference between the same starting conformer charged on Intel processor and an M1 Mac was nearly 0, but there were instances of differences up to 0.03 e.

We find that both AmberTools AM1‐BCC and OpenEye AM1‐BCC partial charge generation methods vary across hardware, given the same input conformer (Figure 3). While the majority of conformers produced similar charges across hardware with average maximal partial charge differences of 0.002 e for AmberTools and OpenEye AM1‐BCC charges, we observe differences of up to 0.09 e using AmberTools AM1‐BCC charges on different hardware platforms and differences of up to 0.025 e using OpenEye AM1‐BCC charges. We confirm that OpenEye AM1‐BCC charges do not differ across repeat charges of the same conformer, but do differ across hardware (Figure S4). However, we find that AmberTools AM1‐BCC charges can differ across repeat charge calculations for the same conformer, so some of this partial charge discrepancy across hardware may come from differences in the convergence of the geometry optimization calculation (Figure S5). We note that OpenEye AM1‐BCC and AmberTools AM1‐BCC partial charge methods do not produce the same partial charges despite being implementations of the “same” AM1‐BCC method, due to implementation details. Specifically, the two approaches at least differ in how they employ geometry optimization before partial charge assignment, as noted above.

**FIGURE 4:**
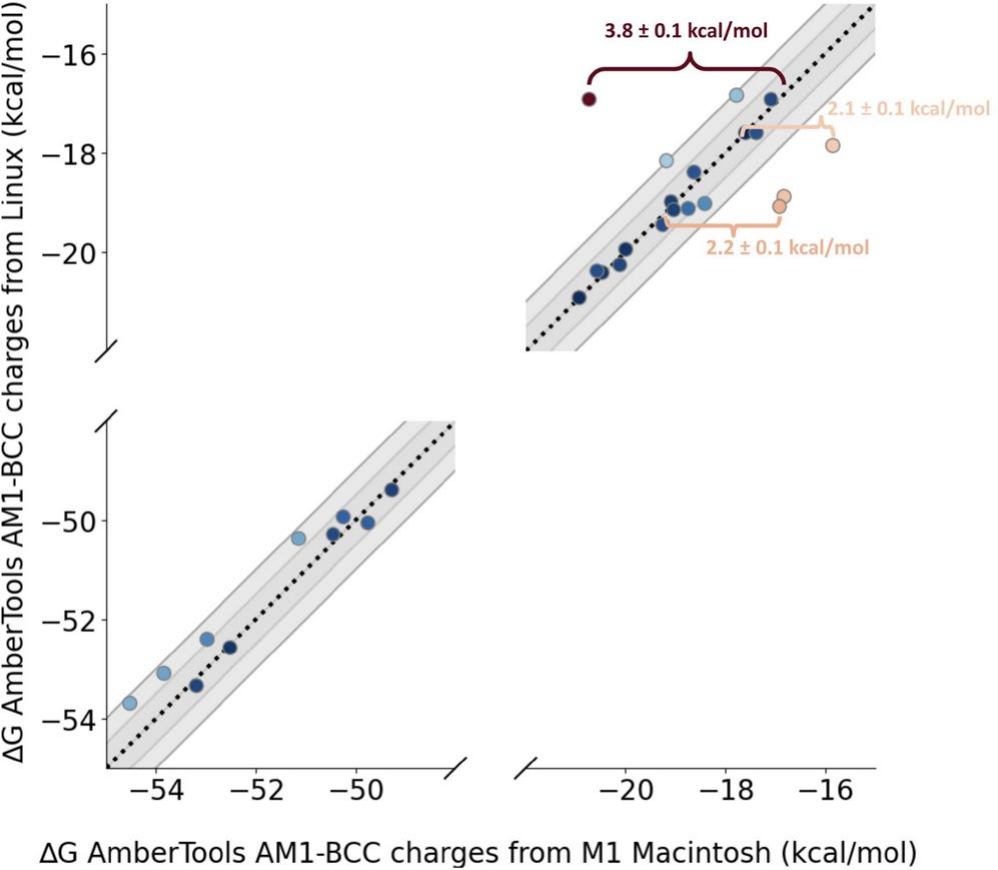
Effects of hardware differences on calculated AHFEs. Each point on this graph represents one repeat per hardware environment for the partial chargers generated from one conformer of a molecule. Variations in calculated values (deviation from the y=x line) are caused by differences in the hardware used for assigning partial charges used for the calculations. For each conformer, we generated partial charges on an Intel processor and an M1 Mac. We ran 2 AHFE calculations for each conformer, where the only difference between the calculations was the partial charges, either from the Intel processor or the M1 Mac. Differences in hardware result in different partial charges given the exact same input conformer. These differences can result in up to 3.8±0.1 kcal/mol differences in calculated AHFE. We note that each conformer only has one repeat per hardware environment used to generate those partial charges. We find that on the timescales simulated in this paper, our free energy estimates from a single partial charge set seem to converge to similar values (Figure S19b,c). On the other hand, free energy estimates from different partial charge sets converge to different values (Figure 5).

We calculate AHFEs for 2 sets of AmberTools AM1‐BCC partial charges generated from the same input conformer, but on different hardware. We find that in most cases, partial charges generated from different hardware yielded similar AHFE values within 1 kcal/mol of each other (Figure 4). However, we observe maximal partial charge differences of 0.09 e, resulting in a 3.8±0.1 kcal/mol difference in calculated AHFE, which is a rather large discrepancy and one which is certainly significant for practitioners. For two of these molecules, we chose to further investigate the source of the change in hydration free energy due to charge variation. In the case of lig_215, we find that most of the free energy change is due to short‐ranged enthalpic interactions (Table S2), and in particular is due to a conformational change. In the case of lig_ejm_43, we find that only a small portion of the free energy change is due to short‐ranged enthalpic contributions, indicating the majority of the difference comes from entropy differences due to the charge model differences (Table S1), We suspect that the reason for the discrepancy will vary for each solute.

### 3.3 Partial Charge Variability Leads to Large Ranges of Calculated ΔG

Calculations such as absolute hydration free energy calculations may be affected by different partial charge sets for a molecule when all other simulation conditions are held constant. We performed absolute hydration free energy calculations on 15 molecules: 5 “bad” molecules, 5 “good” molecules, and 5 “average” molecules. “Bad” molecules are those that had large maximal partial charge ranges. “Good” molecules are those that had small maximal partial charge ranges. For each molecule, we generated 5 partial charge sets from 5 random conformers using AmberTools AM1‐BCC charges. We ran 5 AHFE calculations for each molecule, where each calculation only differed in its assigned partial charges; all other elements, such as starting conformation and coordinates, remained constant across repeats.

**FIGURE 5:**
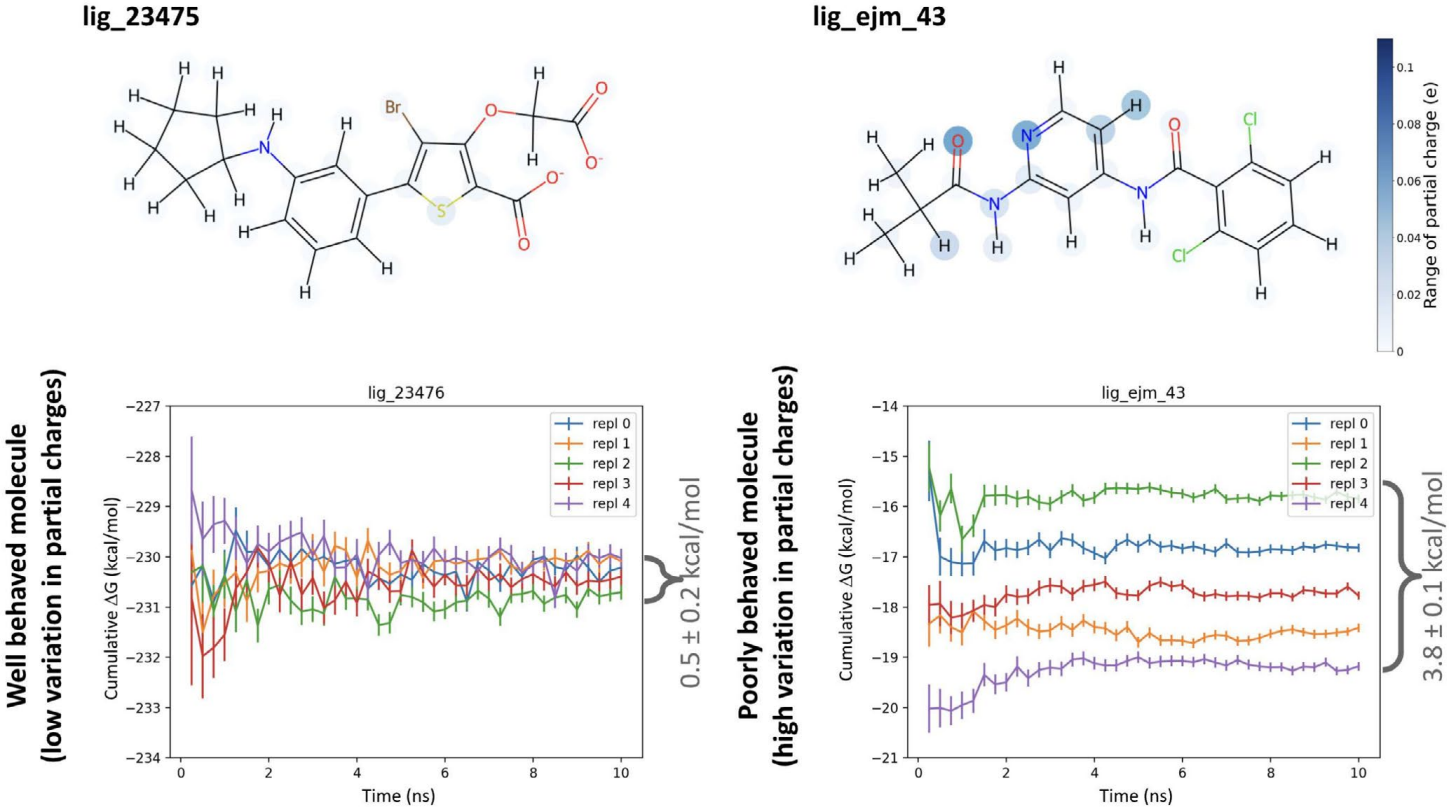
Partial charge variation leads to variable calculated AHFE. lig_23475 is a well‐behaved molecule due to its low variation in partial charges, with a maximal partial charge difference of 0.01 e. AHFE simulations beginning from 5 different partial charge sets of lig_23475 with all other conditions held constant converge to cumulative ΔGs within 0.5±0.2 kcal/mol of each other. lig_ejm_43 is a poorly behaved molecule due to its higher variation in partial charges, with a maximal partial charge difference of 0.06 e. AHFE simulations beginning from 5 different partial charge sets of lig_ejm_43 with all other conditions held constant converge to a larger range of ΔGs within 3.8±0.1 kcal/mol of each other.

In this study, we expected that the more partial charge variability we see across conformers for a molecule, the more discrepancy we would see in calculated ΔG across repeats. We find that AHFE calculation repeats for lig_23475, which has a maximal partial charge difference of 0.01 e, converges to values within 0.5±0.2 kcal/mol (Figure 5). In contrast, repeats of lig_ejm_43, which has a maximal partial charge difference of 0.06e, converge to a much larger range of 3.81 kcal/mol.

Overall, we find that maximal partial charge differences as small as 0.026 e led to calculated ΔG ranges of up to 3.3±0.1 kcal/mol (Table 1). Contrary to our expectations, however, we find that there is seemingly no significant correlation between the magnitude of the hydration free energy (ΔG) and either of the partial charge variation metrics.

**TABLE 1:** Range of calculated AHFE and partial charge differences across 5 partial charge sets for PLB molecules.

	AmberTools AM1‐BCC			OpenEye AM1‐BCC ELF10		
Molecule	Range of ΔG (kcal/mol)	Max partial charge difference (e)	Max Δq_bond difference (e)	Range of ΔG (kcal/mol)	Max partial charge difference (e)	Max Δq_bond difference (e)
lig_23475	0.5±0.2	0.019	0.02	—	—	—
lig_23476	0.7±0.2	0.046	0.071	—	—	—
lig_23477	0.8±0.2	0.016	0.021	—	—	—
lig_p38a_2h	0.9±0.1	0.097	0.142	—	—	—
lig_p38a_2e	1.0±0.1	0.041	0.073	—	—	—
lig_E9	1.3±0.2	0.074	0.078	—	—	—
lig_206	1.4±0.1	0.066	0.083	—	—	—
lig_25	1.5±0.1	0.035	0.044	0.4±0.1	0.006	0.005
lig_215	1.7±0.1	0.034	0.051	0.3±0.1	0.009	0.010
lig_E8	1.7±0.2	0.062	0.063	—	—	—
lig_7	1.9±0.1	0.057	0.096	1.5±0.2	0.016	0.021
lig_E27	2.0±0.1	0.049	0.091	1.5±0.1	0.005	0.095
lig_23472	2.6±0.2	0.058	0.113	—	—	—
lig_ejm_43	3.3±0.1	0.026	0.032	0.8±0.1	0.005	0.006
lig_ejm_31	3.8±0.1	0.055	0.056	1.8±0.1	0.003	0.005

*Note:* The table presents the average calculated AHFE and partial charge differences across various molecules. The values represent the ranges of energetic contributions and differences in charge distributions that impact the molecular behavior in simulations.

### 3.4 Openeye AM1‐BCC ELF10 Charges Resolve Some Issues Related to Conformer‐Dependent Charge Variability

OpenEye AM1‐BCC ELF10 nominally produces charges with the least conformer dependence among available AM1‐BCC methods. To investigate how much OpenEye AM1‐BCC ELF10 charges resolve conformer‐dependent partial charge variability and its effect on variability in calculated AHFE, we chose 6 of the 15 molecules in Section 2.6.2 to study further. We generated 5 sets of OpenEye AM1‐BCC ELF10 partial charges and ran 5‐peats of AHFE calculations, where the only difference between repeats is the set of partial charges used for 6 molecules. We compare these results to the AHFE calculations we ran in Section 3.3.

**FIGURE 6:**
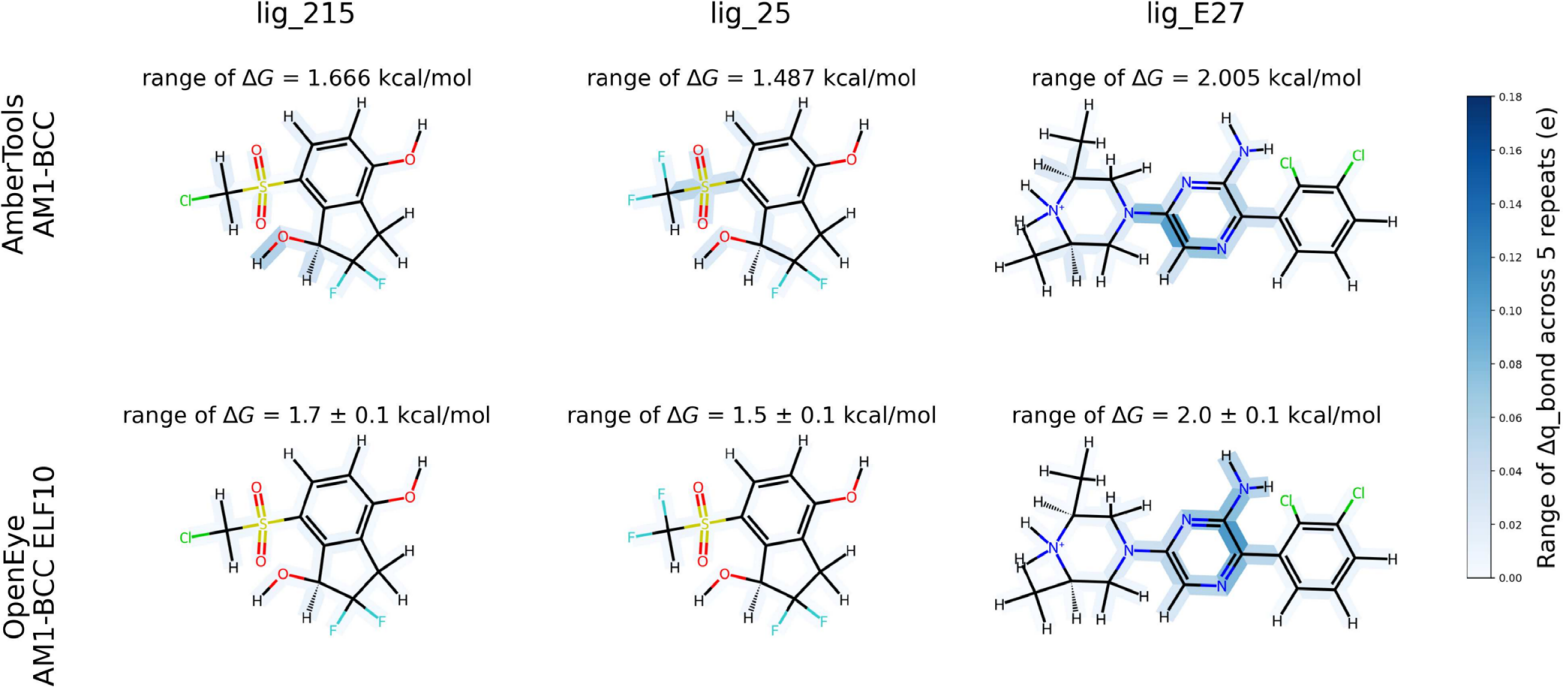
In 2 out of 3 pictured molecules, lig_215 and lig_25, we see smaller ranges of Δq_bond differences when charging with OpenEye AM1‐BCC ELF10 as opposed to AmberTools AM1‐BCC. In these molecules, we see a smaller range of ΔG using the less variable charging method, OpenEye AM1‐BCC ELF10. In lig_E27, OpenEye AM1‐BCC ELF10 and AmberTools AM1‐BCC have a similar maximal range of Δq_bond difference, but OpenEye AM1‐BCC ELF10 charging results in fewer bonds with variability above 0.02 e.

We find that for 5 out of 6 molecules we simulated, AM1‐BCC ELF10 reduces variability in AHFE and both partial charge variability metrics (Table 1). For all 6 molecules, we find that the OpenEye AM1‐BCC ELF10 partial charges reduce the AHFE variability and the maximal partial charge difference. For 5 of the 6 molecules, we find that OpenEye AM1‐BCC ELF10 partial charges reduce the maximal Δq_bond difference (Figure 6).

### 3.5 Conformer‐Independent Open Force Field NAGL Charges Produce Similar Calculated ΔGs to Openeye AM1‐BCC ELF10 With Less Variance

To compare the effect of Open Force Field NAGL charges to OpenEye AM1‐BCC ELF10 charges on calculated ΔG, we calculate AHFEs in 5‐peats for our 6 molecules chosen in Section 3.4 using Openforce field NAGL and compare these to the calculated AHFEs with OpenEye AM1‐BCC ELF10 charges from Section 3.4. Because NAGL charges are conformer‐independent, these 5‐peats have identical starting parameters and thus serve as a sanity check that our AHFE calculations are converging in our 10 ns simulations. If the 5‐peat AHFEs run with NAGL charges converge to similar values, we can assume that 10 ns is satisfactory for converging our AHFE simulations.

**FIGURE 7:**
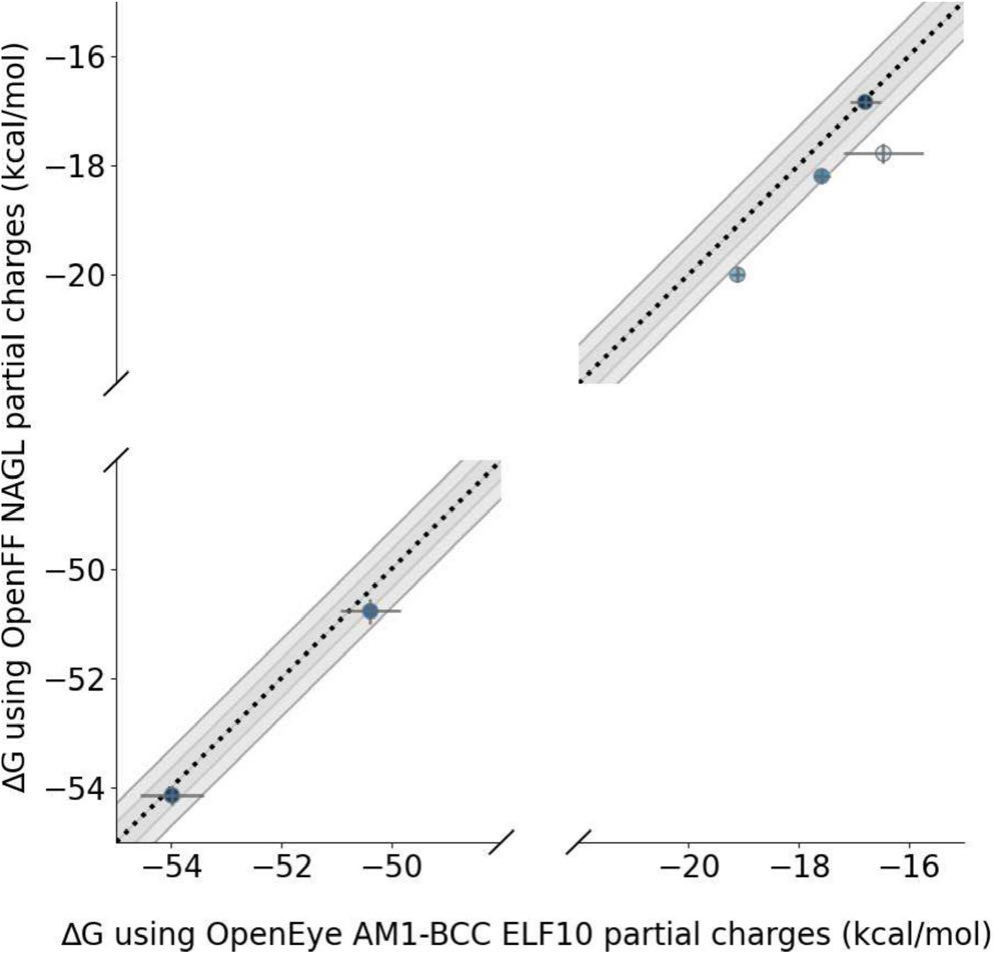
Comparison of calculated AHFE ΔG values using OpenFF NAGL and OpenEye AM1‐BCC ELF10. NAGL and AM1‐BCC ELF10 charges produce similar average ΔG values across repeats; 5 out of 6 molecules are under 1.5 kcal/mol different. Using consistent partial charges, such as through a conformer‐independent method like NAGL, results in less variability across repeats as compared to AM1‐BCC charges alone. However, the variability with NAGL is similar to that observed with the less conformer‐dependent OpenEye AM1‐BCC charges. Since NAGL doesn't have variable charges at all, this indicates a background level of statistical error that is present in all calculations.

We find that OpenEye AM1‐BCC and OpenFF NAGL charges result in similar calculated AHFE values, within 1.5 kcal/mol of one another, for 5 out of 6 molecules (Figure 7). OpenEye AM1‐BCC ELF10 charges, which have conformer dependence, result in more variable calculated AHFEs with standard deviations of up to 0.7 kcal/mol. OpenFF NAGL charges have no variability and result in less variable calculated AHFEs (i.e., all variation comes from statistical error in the hydration free energy calculations themselves) that seem to converge to a single value with standard deviations of 0.2 kcal/mol.

We find that OpenFF NAGL partial charges reduce the variation in calculated AHFE ΔG across 5 repeats as compared to AmberTools AM1‐BCC charges. This is because OpenFF NAGL charges remove one source of error, variable partial charges. We also find that 2 molecules, lig_ejm_31 and lig_ejm_43, have variation greater than 1 kcal/mol in calculated AHFE ΔG using NAGL partial charges, suggesting sampling issues contributed to the variation in these molecules (Table 2).

**TABLE 2:** Range of calculated AHFE across 5 repeats using AmberTools AM1‐BCC, OpenEye AM1‐BCC ELF10, and OpenFF NAGL partial charges for PLB molecules.

Molecule	Range of ΔG AmberTools AM1‐BCC (kcal/mol)	Range of ΔG OpenEye AM1‐BCC ELF10 (kcal/mol)	Range of ΔG OpenFF NAGL (kcal/mol)
lig_25	1.5±0.1	0.4±0.1	0.2±0.1
lig_215	1.7±0.1	0.3±0.1	0.6±0.1
lig_7	1.9±0.1	1.5±0.2	1.2±0.2
lig_E27	2.0±0.1	1.5±0.1	0.7±0.1
lig_ejm_43	3.3±0.1	0.8±0.1	1.3±0.1
lig_ejm_31	3.8±0.1	1.8±0.1	1.0±0.1
Average	2.4±0.3	1.05±0.3	0.8±0.3

### 3.6 Case Study: MobleyLab/FreeSolv

To further study the effect of conformer‐dependent partial charges on calculated AHFEs, we mine FreeSolv for 12 molecules with variable Δq_bond differences. We run 5‐peats of AHFE calculations for these molecules, where the only difference between repeats is the partial charge set employed. We compare the calculated AHFE for each repeat back to experimental hydration free energy data.

We find that the FreeSolv molecules also display conformer‐dependent partial charge variability that affects our AHFE calculations despite having fewer rotatable bonds than the PLB molecules. The FreeSolv molecules vary in maximal partial charge difference by up to 0.493 e and in maximal Δq_bond difference by up to 0.835 e (Table 3). We see discrepancies of up to 6.9±0.1 kcal/mol across the five repeats. We find that our AHFE calculations tend to agree with previous FreeSolv calculations in 9 out of 12 cases (Figure S17). Comparison with experimental results can also be of interest, though we expect agreement with experiment not to be ideal due to the underlying force field not completely capturing reality. Thus, in comparison to the experimental hydration free energies for the FreeSolv molecules, we find a root mean squared error (RMSE) from experiment of 3.8±0.6 kcal/mol. We were also interested in determining whether NAGL gave results of at least comparable accuracy, so we ran a single replicate of these same calculations using OpenFF NAGL charges (Figure S17). We find that hydration free energies computed with OpenFF NAGL charges give a similar level of agreement with experimental AHFE values, with an RMSE from experiment of 3.3±0.6 kcal/mol (Figure S22).

**TABLE 3:** Range of calculated AHFE across 5 conformers using AmberTools AM1‐BCC partial charges for FreeSolv molecules.

Molecule	Range of ΔG AmberTools AM1‐BCC (kcal/mol)	Maximal partial charge difference (e)	Maximal $\Delta q_{\text{bond}}$ difference (e)
$mobley_{\text{\_}}1944394$	0.04±0.09	0.036	0.04
$mobley_{\text{\_}}3201701$	0.2±0.1	0.017	0.027
$mobley_{\text{\_}}8558116$	0.37±0.06	0.026	0.025
$mobley_{\text{\_}}9653690$	0.38±0.07	0.047	0.05
$mobley_{\text{\_}}3047364$	0.5±0.1	0.027	0.044
$mobley_{\text{\_}}6338073$	0.7±0.1	0.048	0.063
$mobley_{\text{\_}}7688753$	0.69±0.07	0.056	0.062
$mobley_{\text{\_}}8916409$	1.4±0.1	0.093	0.125
$mobley_{\text{\_}}7326706$	1.5±0.1	0.094	0.126
$mobley_{\text{\_}}1770205$	3.3±0.1	0.176	0.263
$mobley_{\text{\_}}1849020$	6.5±0.1	0.681	0.835
$mobley_{\text{\_}}9460824$	6.9±0.1	0.493	0.593

## 4 Discussion

Calculated free energies are dependent on the partial charges assigned to the molecules involved. Using different partial charge methods, different hardware for generating partial charges, and different conformers of a molecule can all result in variable partial charge assignments for the same molecule. While conformer‐dependent effects on partial charge assignment are expected due to the nature of the wave function, hardware‐dependent effects are likely due to numerical issues in the software implementations of partial charge methods. Both of these issues lead to difficulties in reproducing calculated binding free energies and hydration free energies (AHFEs) as well as experimental hydration free energy values across different partial charge sets of the same molecule.

### 4.1 Different Partial Charge Sets Lead to Quite Different Calculated Values

In practice, assigned partial charges can have significant impacts on downstream free energy calculations. We find that differences in partial charges as small as 0.026 e can result in large ranges in calculated AHFE of up to 3.3±0.1 kcal/mol. This means that partial charge methods that can reproduce partial charge sets for a molecule with maximal partial charge differences of ≤0.03 e can still lead to poor reproducibility of free energy calculations.

We suspect partial charges are the dominant source of error in our calculation repeats. In running 5 identical repeats where each repeat had the same partial charges, we find the maximal range of calculated free energy out of 6 molecules to be 1.2±0.2 kcal/mol with an average range across 6 molecules of 0.8±0.3 kcal/mol, indicating that sampling error/convergence led to variation of only 0.6±0.1 to 1.2±0.2 kcal/mol. However, when we run 5‐peats of the same 6 molecules where each repeat only differs by the partial charge set used, we find the maximal range of calculated free energy out of 6 molecules to be 3.8±0.1 kcal/mol with an average range across 6 molecules of 2.4±0.3 kcal/mol. All 6 molecules were able to converge to within 1.2±0.2 kcal/mol of each other across 5‐peats using the same partial charge sets across repeats. When using differing partial charge sets across repeats, we see much larger ranges of calculated free energies across repeats. For a particular system, force field (including charge model) and set of simulation parameters, a converged free energy calculation ought to yield (within statistical error) a single free energy for that particular model; this single free energy could be considered a gold standard value for that particular application. Compared to this reference gold standard value, variable partial charges induce errors in free energy calculations and corresponding deviations from the gold standard value. Here, we find these variations dominate over other common sources of error, such as sampling errors.

### 4.2 Methods With Lesser Conformation Dependence Do Not Always Fix the Problem

We investigate whether OpenEye AM1‐BCC ELF10 charges mitigate the conformational dependence of partial charges. We selected 6 out of the 15 molecules chosen for AHFEs that had large ranges of calculated AHFEs across partial charge sets to study further. In 5 out of 6 molecules, we see ELF10 charges decrease the conformer dependence on partial charge generation as compared to AmberTools AM1‐BCC. However, in the 6th molecule, lig_E27, we see no improvement in partial charge variability when moving from AmberTools AM1‐BCC to OpenEye AM1‐BCC ELF10 charges. ELF10 charges alleviates the problem of variable partial charges due to conformer dependence some of the time, but it is not a guaranteed fix. Moving to less conformer‐dependent charges, such as AM1‐BCC ELF10, can thus provide *some* mitigation of partial charge variability. In all 6 molecules, AM1‐BCC ELF10 charges reduced charge variability with both metrics. There is still some conformer dependence, and even small differences in partial charges of 0.026 e can result in discrepancies of 3.1±0.1 kcal/mol in calculated free energies (Table 1). Removing all conformer‐derived partial charge variability using OpenFF NAGL charges results in calculated free energies that still vary slightly due to the background level of statistical error affecting these calculations, exhibiting roughly the same level of variability as with AM1‐BCC ELF10 charges.

### 4.3 Hardware is Another Source of Partial Charge Variability

We investigate the effect of variable hardware on calculated partial charges. We find that hardware can produce maximal partial charge differences of up to 0.09 e and up to 3.8±0.1 kcal/mol discrepancies in calculated AHFEs. This means that even given the same input conformer for an AHFE and the exact simulation settings, a researcher may not be able to reproduce another researcher's work unless partial charge values are shared. While the AM1‐BCC charge method should be deterministic and not change with multiple runs of the same conformer, we do not find this to be the case. The present findings strongly suggest that the authors of both examined AM1‐BCC implementations ought to check their work to ensure stable convergence and robustness across variations in architecture.

### 4.4 Freesolv Molecules Exhibit Large Conformer‐Induced Partial Charge Variability and Resulting Variability in Calculated Free Energies

Lastly, we investigate the effect of partial charge variability on our calculated AHFEs with a set of 11 FreeSolv molecules, which we can also compare back to experiment. We find that the FreeSolv molecules are still subject to maximal partial charge variabilities of up to 0.681 e, resulting in ranges of ΔGs of up to 6.9±0.1 kcal/mol. Despite these molecules being smaller and having fewer unique conformers, we still see significant differences in partial charge and calculated AHFE. We confirmed these values were due to the partial charges rather than convergence errors by running 2 more repeats of the partial charge sets that led to the minimum and maximum calculated ΔG values. We found the level of calculated ΔG variation remained constant across these additional repeats (Figure S19). This suggests that even relatively small molecules are subject to partial charge reproducibility concerns.

### 4.5 Conformer‐Dependent Charges Pose Problems for Reproducibility in a Fixed‐Charge Framework

Within a fixed‐charge model, if charges are derived from quantum chemical approaches, as they have been in many present‐day fixed‐charge classical force fields, it is reasonable that the resulting charges should have some dependence on the choice of conformer used for charging. Specifically, both the HF/6‐31G* RESP charge model [30] and the AM1‐BCC semi‐empirical framework, which is trained to be somewhat like it [49], quite reasonably result in conformer‐dependent charges. Shouldn't the charges used for downstream modeling (such as that done here) thus exhibit significant conformer dependence? In other words, quantum chemistry is conformer‐dependent.

In our view, there is an important distinction between conformer‐dependent charges in this sense (that quantum chemistry assigns charges that depend on the choice of conformer) and what one might call “user‐dependent” charges, which depend on the specific choice of input conformer, charge assignment algorithm implementation, and hardware platform. In the area of binding and solvation thermodynamics, users want to (and ought to be able to expect) obtain reliable, reproducible free energy estimates without having to use the same compute platform and agree on which conformer of a molecule to provide as user input.

Here, our results show that variation in input conformer, details of charge assignment algorithm implementation, and hardware platform can adversely affect charge assignment reproducibility in a way that has a profound impact on the accuracy and reproducibility of downstream free energy estimates. This will likely lead to increased interest in charge models, which are conformer‐independent.

At some level, of course, charges *ought* to be conformer‐dependent in the sense that the underlying electronic structure of a molecule depends on its conformer. However, this electronic structure is not possible to treat within a classical fixed‐charge model, as charges would need to rearrange with the molecule and this rearrangement would need its energetics modeled—necessitating the use of a Hamiltonian to describe these charge fluctuations (such as a polarizable or fluctuating charge model [50, 51, 52]). Within the classical fixed‐charge framework, then, the solution is to use a single set of fixed charges—often, a set derived from an optimal or representative conformer, or one obtained from averaging across conformers. Multiple approaches are possible, but the goal is to use a single representative set of charges that works well across all relevant geometries of the molecule. Here, our results indicate that current implementations of AM1‐BCC do not yet provide such representative charges, so further improvements are needed.

We suspect that these reproducibility problems will persist in more complex pharmaceutically relevant calculations, such as binding free energy calculations. The thermodynamic cycle for solvation free energies involves a gas‐to‐water transfer free energy (two phases), both of which are affected by the choice of partial charges, so the present work already observes a lack of cancellation across sides of the thermodynamic cycle. If the errors induced by different partial charge sets canceled between the two phases, we would not see discrepancies as large as 3.8±0.1 kcal/mol across the present calculations. Thus, in protein‐ligand binding free energy calculations, we expect the same issues will persist, as supported by the preliminary Open Free Energy relative binding free energy data (Figure 1).

In the case of both hydration free energies and binding free energies, we assume that an optimal set of charges from a classical fixed‐charge model is adequate; there is simply no framework for allowing partial charge variation within a classical fixed‐charge model such as those employed here. To move beyond this assumption, a different framework (such as a polarizable model) would need to be employed.

## 5 Conclusion

Overall, we have demonstrated that conformer‐dependent charge assignment methods can have a functionally important effect on calculated absolute hydration free energy values with ranges of up to 6.9±0.1 kcal/mol from only input conformer differences. Using the same input conformer and charge assignment methods but different hardware can result in calculated absolute hydration free energy values with ranges of up to 3.8±0.1 kcal/mol. This means that binding free energy studies, which have even more degrees of freedom and sources of variability, will suffer from unpredictable performance introduced by variation in the partial charge set. These results thus highlight the importance of using reproducible partial charges that are relevant to the problems or questions we are trying to address in our simulations. The partial charge variations observed here may be remedied by using conformer‐independent charges or by investing more research into determining how to unambiguously assign reliable charges regardless of input conformer. Otherwise, what is essentially noise can be triggered by variations in user input conformations, conformer‐generation engine, or even choice of compute hardware, impeding the reproducibility of binding free energy calculations even when done with the “same” force field, method, and tools, thus adversely affecting both predictive accuracy and method development and testing.

## Author Contributions

**Meghan Osato:** set up and ran simulations, collected all partial charge and solvation free energy data. Coordinated the paper, contributed to/drafted all sections, and designed all figures. **Hannah M. Baumann:** contributed free energy data for Figure 1. Co‐designed the solvation free energy protocol. Contributed to the outline, edited and reviewed text and figures. **Jennifer Huang:** contributed partial charge distribution data for HIF2a truncated dataset. **Irfan Alibay:** contributed free energy data for Figure 1. Co‐designed the solvation free energy protocol. Contributed to the outline, edited and reviewed text and figures. **David L. Mobley:** supervised the work, contributed to the outline, edited and reviewed text and figures.

## Conflicts of Interest

David L. Mobley serves on the scientific advisory boards of Anagenex and OpenEye Scientific Software, Cadence Molecular Sciences. He is also an Open Science Fellow with Psivant.

## Supporting information


**Data S1.** Supporting Information.

## Data Availability

All code, data, and results from this study are made freely available and can be found on GitHub at https://github.com/mobleyLab/ConformerDependentCharges. We also include these as tarball archives in our submission, but any subsequent updates can be found at the above GitHub repository. The data that supports the findings of this study are available in the Supporting Information of this article.
