# Supplementary material for: Evaluating the Functional Importance of Conformer‐Dependent Atomic Partial Charge Assignment
Source: J Comput Chem. 2025 May 14;46(13):e70112. doi: 10.1002/jcc.70112 (PMC12079016; doi:10.1002/jcc.70112)
Supplement: Supplementary file 1 — Data S1. Supporting Information. [file JCC-46-0-s001.pdf]

6 Supplementary Information

Evaluating the functional importance of conformer-dependent atomic partial charge assignment

Meghan Osato[1], Hannah M. Baumann[2], Jennifer Huang[1], Irfan Alibay[2], David L. Mobley[1,2,*]

[1]Department of Pharmaceutical Sciences, University of California, Irvine, CA 92697, USA

[2]Open Free Energy, Open Molecular Software Foundation, Davis, 95616 California, United States.

[3]Department of Chemistry, University of California, Irvine, CA 92697, USA

*Corresponding Author

Email: dmobley@mobleylab.org

6.1 Truncation of HIF2a molecules still results in conformer partial charge variability

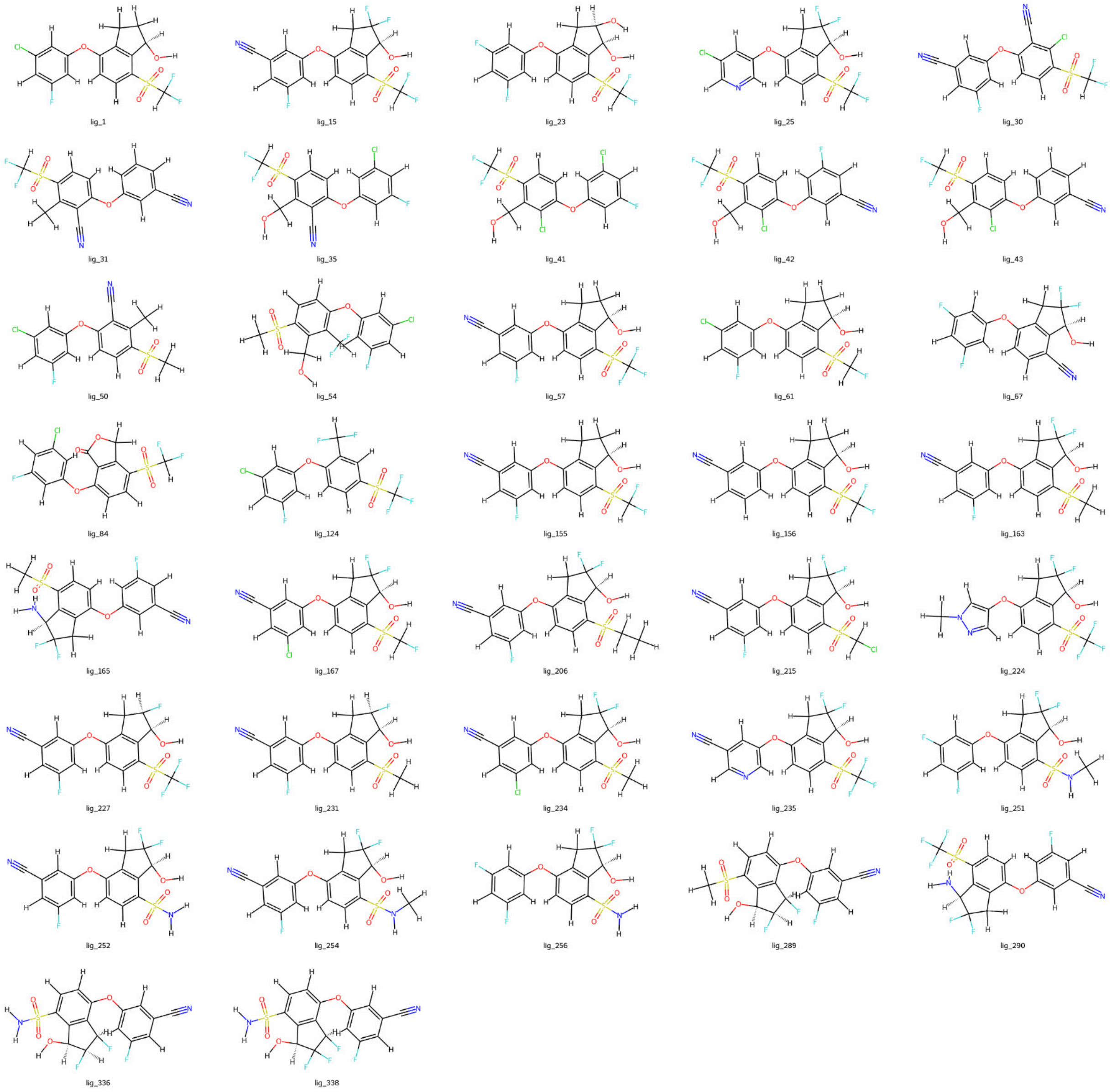

Figure S1: HIF2a whole molecules. Here we show 2D depictions of the unmodified molecules from the HIF2a PLB set

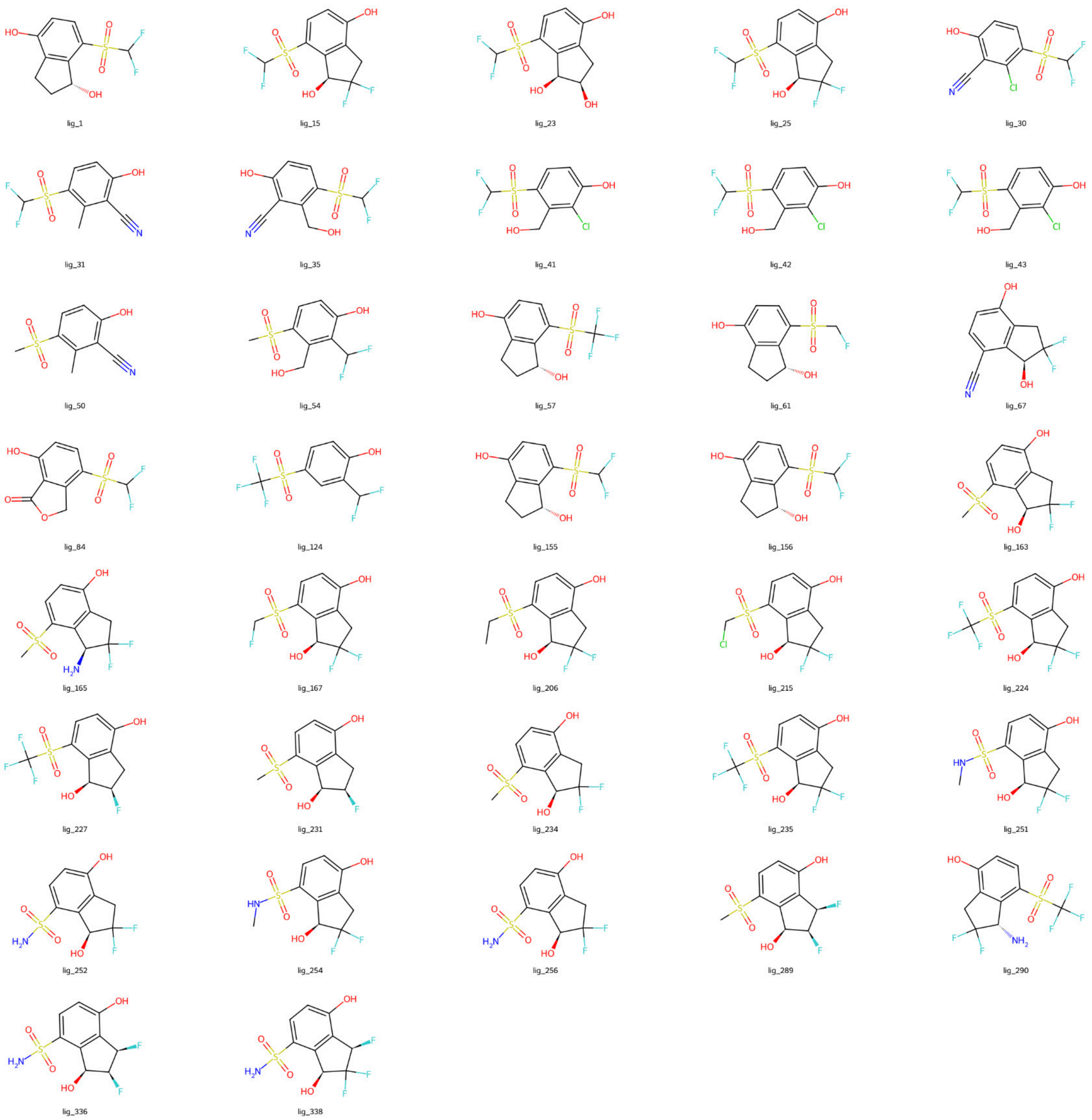

Figure S2: HIF2a truncated molecules. Here we show 2D depictions of the truncated molecules from the HIF2a PLB set

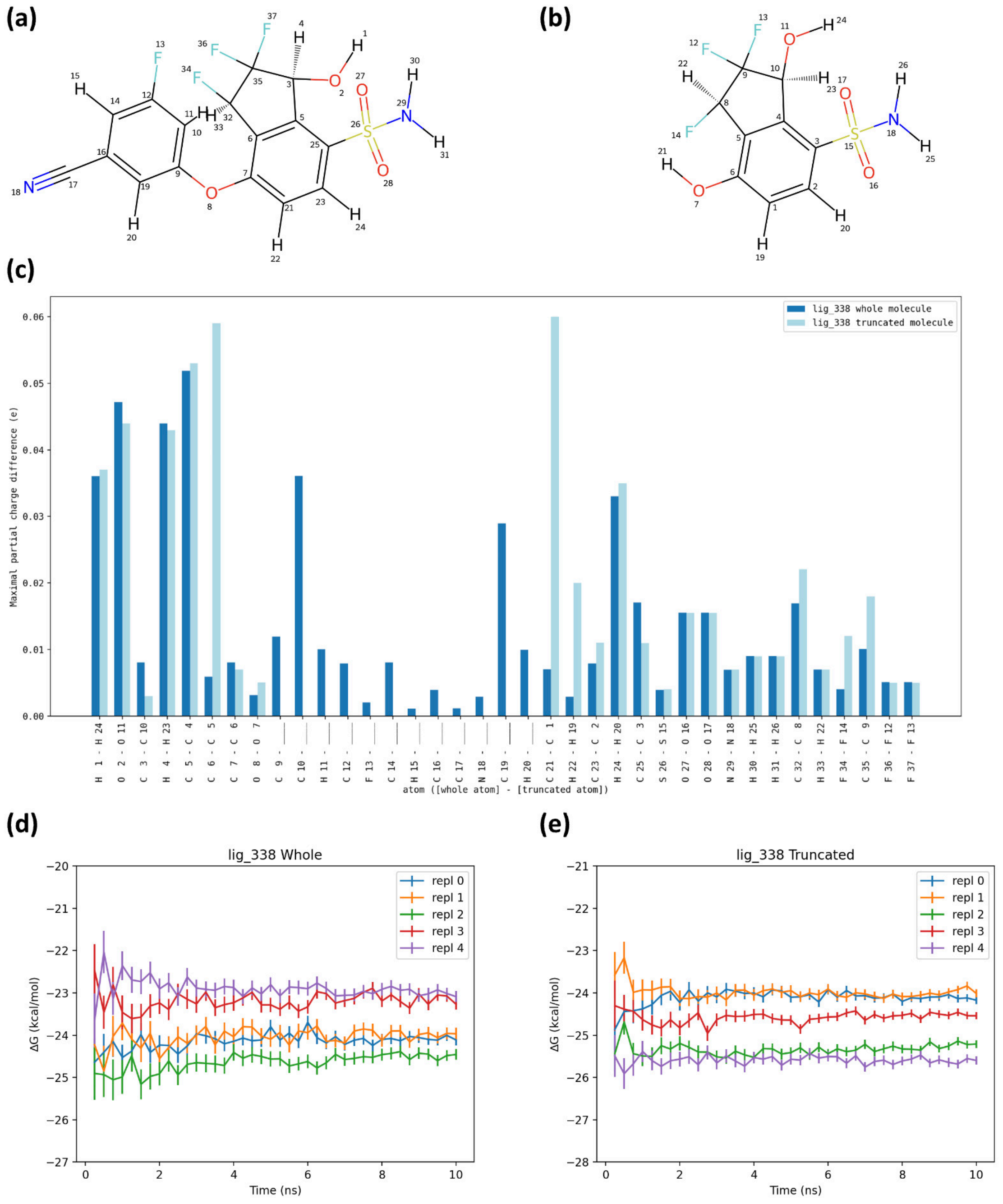

Figure S3: Truncation of HIF2a molecules does not affect partial charge variability nor free energy variability. **(a)** HIF2a *lig*_338 whole molecule with atom indices labeled. **(b)** HIF2a *lig*_338 truncated molecule with atom indices labeled. **(c)** Maximal partial charge difference across 5 random rdkit conformers between the whole and truncated molecules is comparable. The whole molecule has a maximal partial charge difference of approximately 0.05 e. The truncated molecule has a maximal partial charge difference of approximately 0.06 e. **(d)** We ran absolute hydration free energy calculations for 5 replicates of the *lig*_338 whole molecule where only the partial charges differed. We saw a ΔG range of 1.02 ± 0.01 kcal /mol. **(e)** We ran absolute hydration free energy calculations for 5 replicates of the *lig*_338 truncated molecule where only the partial charges differed. We saw a ΔG range of 1.6 ± 0.1 kcal/mol.

6.2 Partial charges vary with the hardware they were generated on

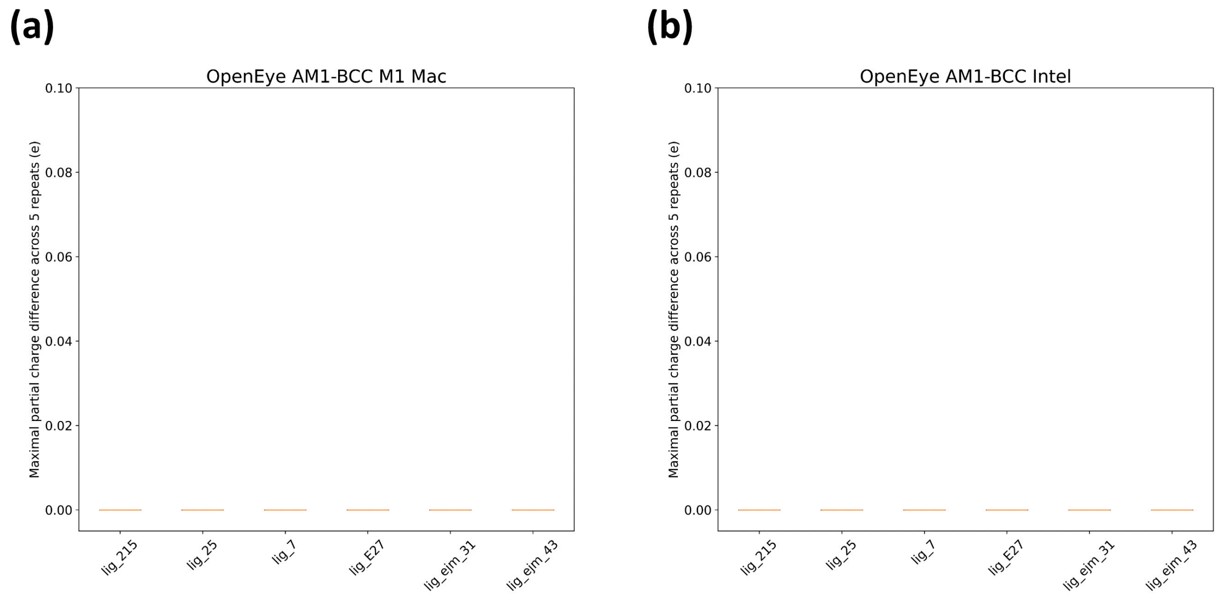

Figure S4: OpenEye AM1-BCC charges do not differ across multiple trials of the same conformer. Here, we generate 50 conformers of the 6 PLB molecules chosen for simulation. We charge each conformer 5 times, and take the maximal partial charge difference across the five trials. We then plt the maximal partial charge difference seen across the 5 trials a conformer for each molecule as a box and whisker plot. **(a)** OpenEye AM1-BCC charges do not differ when the same input conformer is provided on an M1 Mac processor. **(b)** OpenEye AM1-BCC charges do not differ when the same input conformer is provided on an Intel processor.

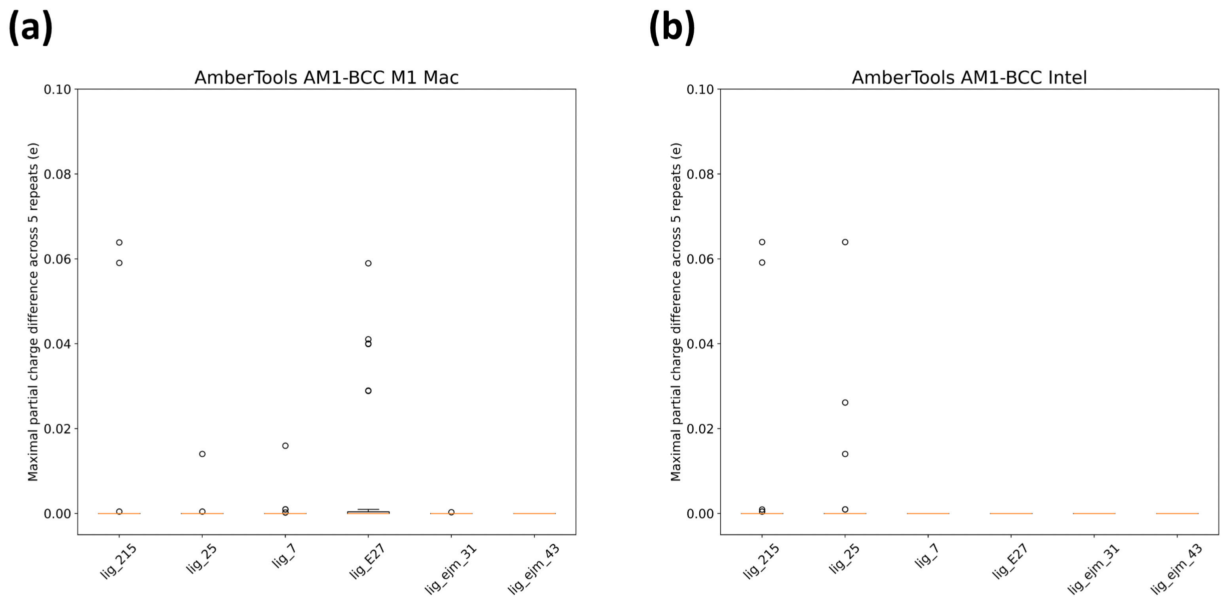

Figure S5: AmberTools AM1-BCC charges differ across multiple trials of the same conformer using the default SQM settings. Here, we generate 50 conformers of the 6 PLB molecules chosen for simulation. We charge each conformer 5 times, and take the maximal partial charge difference across the five trials. We then plot the maximal partial charge difference seen across the 5 trials of a conformer for each molecule as a box and whisker plot. **(a)** AmberTools AM1-BCC charges differ when the same input conformer is provided on an M1 Mac processor. **(b)** AmberTools AM1-BCC charges differ when the same input conformer is provided on an Intel processor.

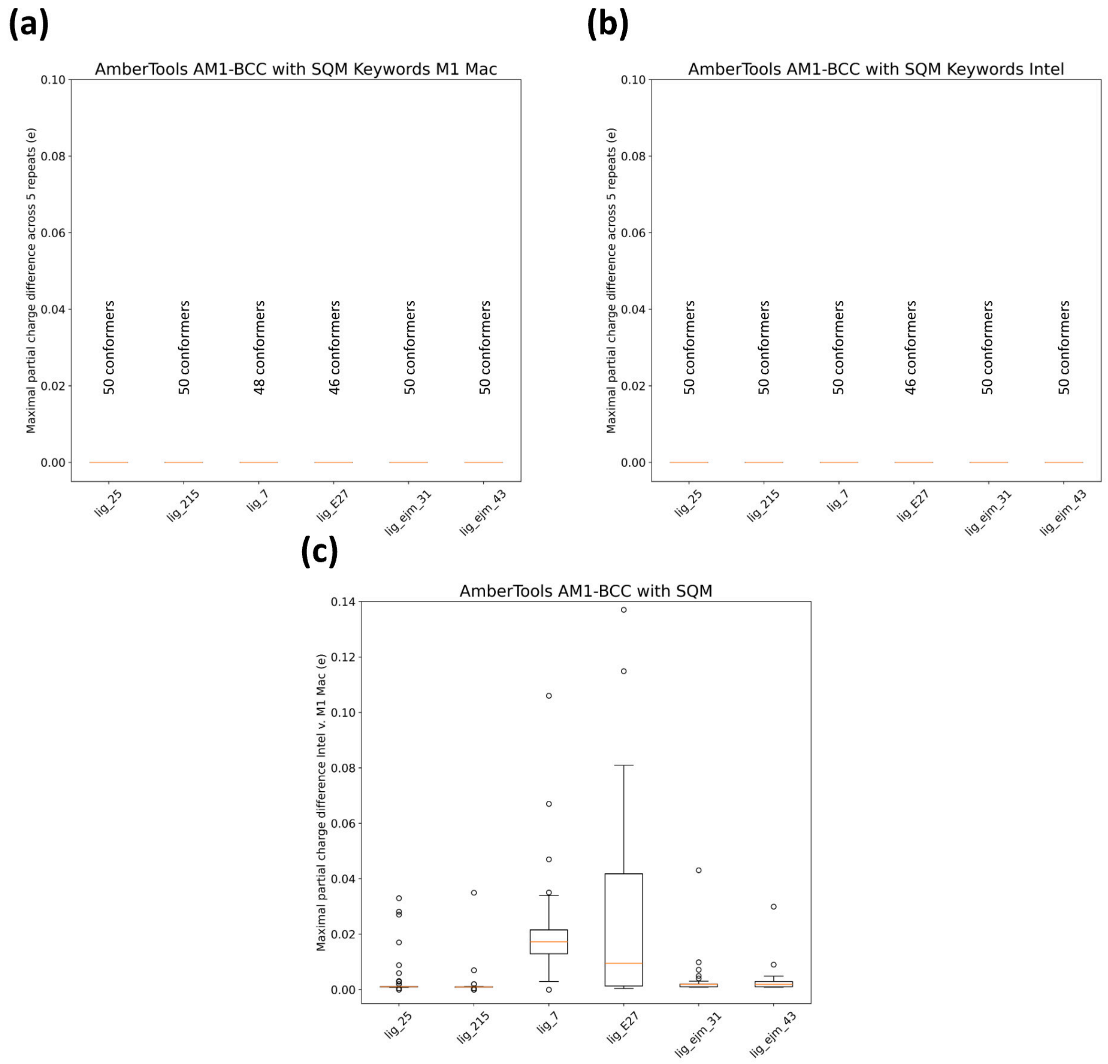

Figure S6: AmberTools AM1-BCC charges are consistent multiple trials of the same conformer when SQM keywords are altered from default. After noting that AmberTools AM1-BCC charges differ across multiple trials of the same conformer using the default settings (SI Figure S5), we confirm that this difference is caused by SQM convergence issues. We modify the SQM keywords: $pseudo\_diag = 0$, to force full diagonalization and $diag\_routine = 1$, to use the internal diagonalization routine. When these keywords are modified we note that the partial charges are consistent across multiple charges of the same conformer. **(a)** AmberTools AM1-BCC charges from an M1 Mac processor are consistent when we provide the same conformer and the above SQM keywords. We exclude two conformers of lig_25 and 4 conformers of lig_E27 as they did not converge with the SQM keywords. **(b)** AmberTools AM1-BCC charges from an Intel processor are consistent when we provide the same conformer and the above SQM keywords. We exclude 4 conformers of lig_E27 as they did not converge with the SQM keywords. **(c)** We find that even with the SQM keywords and the same conformers as input, there is still a discrepancy in partial charges generated from M1 Mac and Intel processors. This suggests that the SQM keywords used will not fix conformer dependent charges. We exclude two conformers of lig_25 and 4 conformers of lig_E27 as they did not converge with the SQM keywords for both processors.

6.3 Correlation between partial charge variability metrics and magnitude of calculated absolute hydration free energy difference across repeats

6.3.1 Partial charge variability is correlated with variability in the solvent annihilation step calculated ΔG

We assess the relationship between variability in partial charges and resulting variability in calculated ΔGs. In a study by the Open Free Energy, variability in calculated ΔG due to variable partial charges was previously observed in relative binding free energies (RBFE). However, because RBFEs are expensive calculations, we investigate whether similar effects would appear in simpler AHFEs, allowing for a more rapid and systematic study. We find the variation calculated ΔG persisted in absolute hydration free energies (AHFE), so the remainder of this study focuses on these calculations.

We generate 5 conformers of each molecule, and charge them using AmberTools AM1-BCC charges, resulting in 5 different sets of partial charges. We run 5-peats of AHFEs for each molecule where each repeat varies only the partial charge set and holds all other details constant, including starting conformer, starting positions, etc. If variable partial charges have little or no effect on AHFE calculations, we expect that there will be little variation in our calculated AHFE ΔGs across 5-peats. To assess the magnitude of the effect variable partial charges our calculated ΔGs, we investigate the relationship between the ΔG of the solvent leg, sometimes referred to as the solvent annihilation step, of our AHFE and its relationship to our 2 partial charge variability metrics, maximal range of partial charge and maximal range of $\Delta q\_bond\ difference$. We use $\Delta G_solvleg$, read as "ΔG of the solvent leg", to describe the effect of partial charge variability on calculated free energy. We do this rather than using the cycle closed, final AHFE ΔG value as this can mask errors due to errors canceling when the free energy cycle is closed.

We find that both metrics – maximal range of partial charge and maximal range of

$\Delta q\_bond\ difference$ – are positively correlated with variability of $\Delta G_{solvleg}$ across 5-peats (Figure S7). We refer to the variability of $\Delta G_{solvleg}$ as the range of $\Delta G_{solvleg}$, the range of $\Delta G_{solvleg}$ we see across all 5 repeats. We find that the variability in $\Delta G_{solvleg}$ is comparable to the variation we see in the corresponding $\Delta G_{vacuumleg}$. Our maximal range of partial charge metric is weakly correlated with the range of $\Delta G_{solvleg}$ across 5-peats with R^2=0.49 (Figure S7a). Our maximal range of $\Delta q\_bond\ difference$ metric is correlated with the range of ΔG solvent leg across 5-peats with R^2=0.58 (Figure S7c). We find that even small differences in partial charge and $\Delta q\_bond\ differences$ of <0.01 e can lead to variations in $\Delta G_{solvleg}$ of 8.9 ± 0.1 kcal/mol and variations in ΔG of 1.7 ± 0.2kcal/mol (Figure S7a,c).

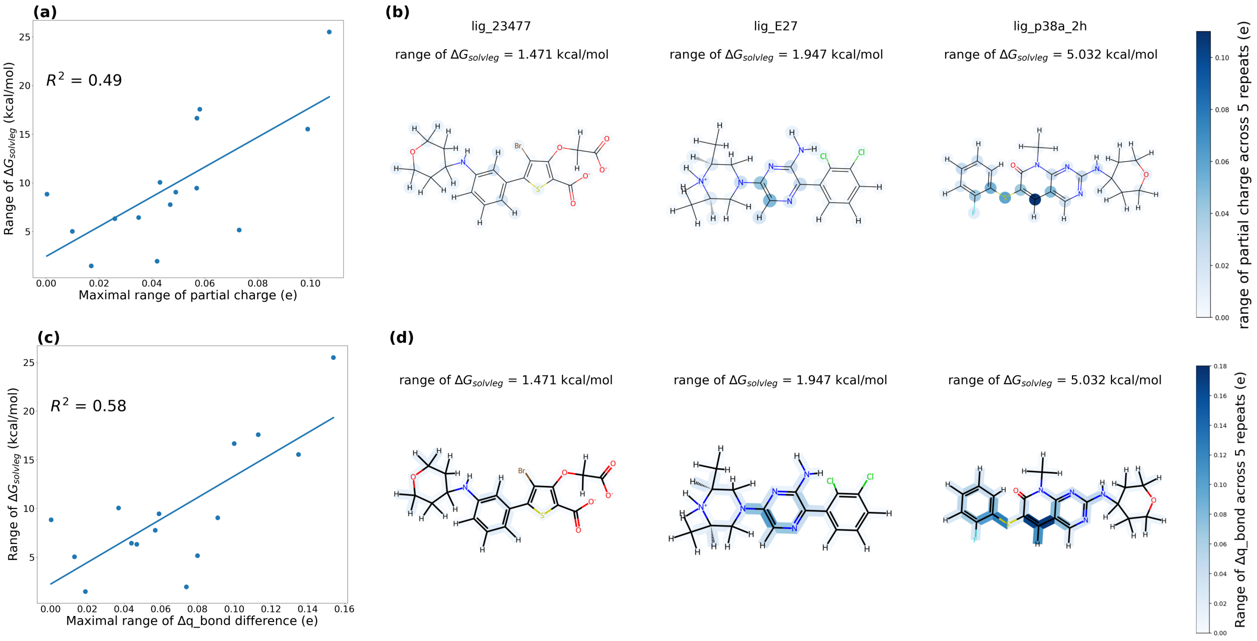

Figure S7: Predictive power of partial charge variability metrics, maximal range of partial charge and Maximal range of $\Delta q\_bond\ difference$. **(a)** Maximal range of partial charge is a weakly correlated with variability in solvent leg ΔG ($\Delta G_{solvleg}$) with an R^2 of 0.49. **(b)** Examples of molecules that have low, moderate and high charge variability based on the Maximal range of partial charge metric. **(c)** Maximal range of partial charge is correlated with variability in solvent leg ΔG ($\Delta G_{solvleg}$) with an R^2 of 0.58. **(d)** Examples of molecules that have low, moderate and high charge variability based on the maximal range of $\Delta q\_bond\ difference$ metric. Maximal range of $\Delta q\_bond\ differences$ seen across conformers has higher predictive power with respect to ΔG variability than the maximal range of partial charges seen across conformers.

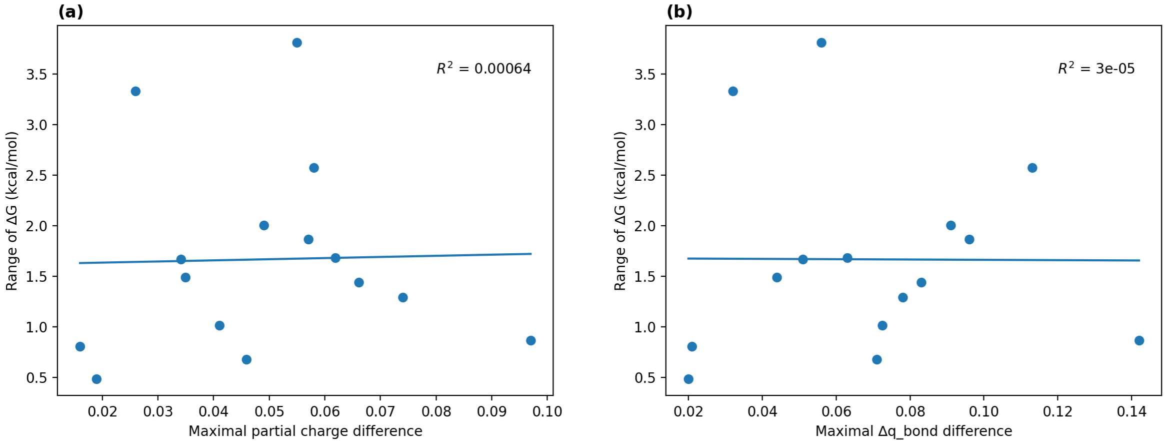

Figure S8: Correlation between range of cycle closed ΔG partial charge difference metrics. **(a)** We found no correlation between the maximal partial charge difference metric and the range of cycle closed ΔG. **(b)** We found no correlation between the maximal Δ*q_bond difference* metric and the range of cycle closed ΔG.

6.4 ΔG versus time plots suggest our calculations converge within 10ns

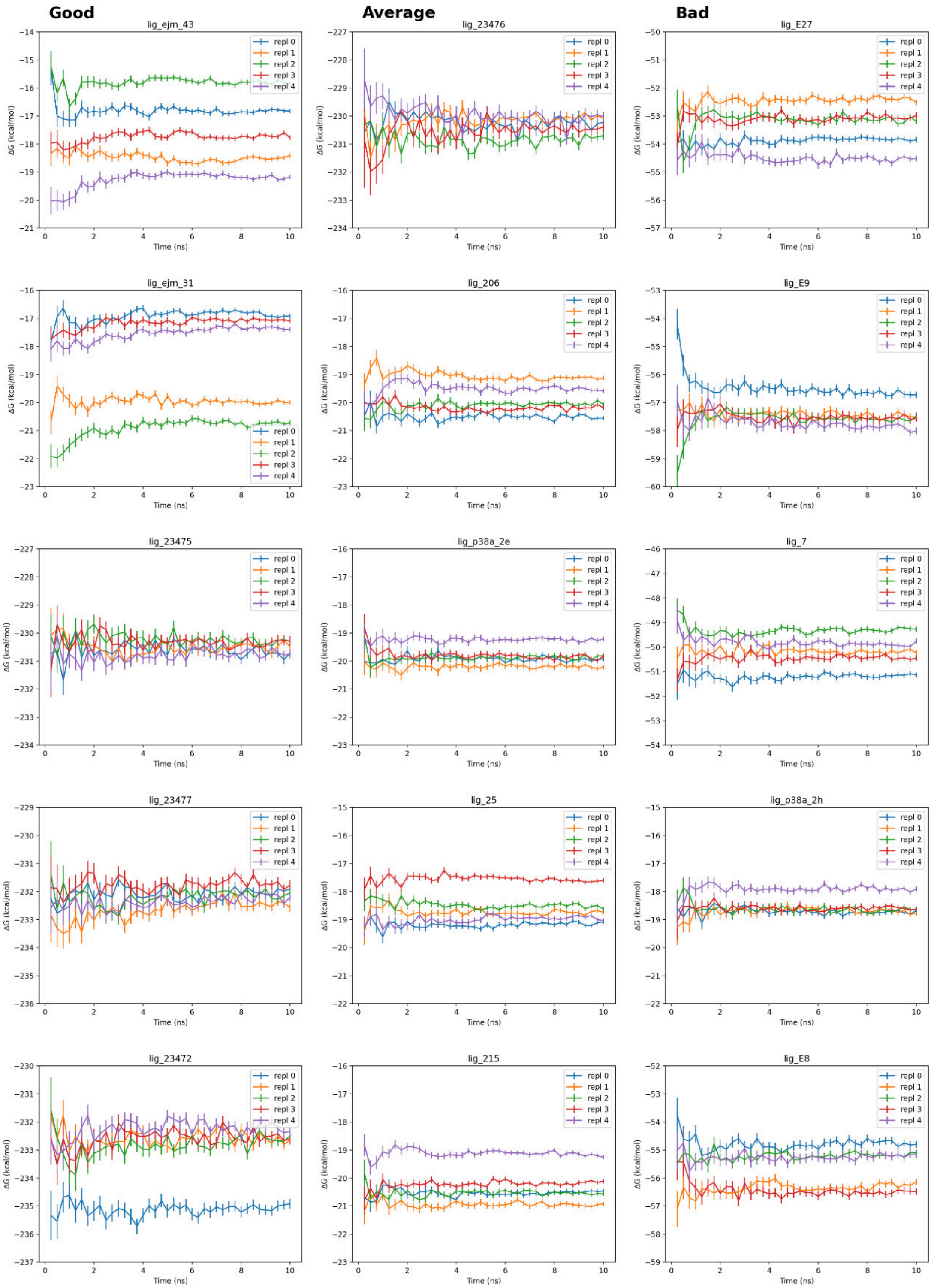

Figure S9: PLB AHFE ΔG versus time using AmberTools AM1-BCC charges from an M1 Mac processor. We ran AHFE calculations for 15 molecules of the PLB dataset. Each column represents the 5 molecules simulated from each category: "good", "average", and "bad". Each plot shows 5 replicates, each starting from a different partial charge set. We find that all simulations trials seemingly converge as their ΔG versus time plots flatten out.

Table S1: *lig_215* End-state energy analysis for Replicate 0 and 3 in solvent and vacuum

Interaction	Replicate 0 (kJ/mol)	Replicate 3 (kJ/mol)	Energy Difference (kJ/mol)
Solvent LJ-SR Solvent-Solute	-63.148	-63.402	0.254
Solvent LJ-SR Solute-Solute	-11.159	-10.936	-0.223
Solvent Coulomb-SR Solvent-Solute	-190.898	-192.714	1.816
Solvent Coulomb-SR Solute-Solute	-205.109	-201.659	-3.45
Vacuum LJ-SR	-9.789	-7.839	-1.949
Vacuum Coulomb-SR	197.944	192.258	-5.686

To examine the mechanism of *how* charge variations introduce such substantial free energy changes, we ran end-state simulations for two different ligands. In particular, we ran such simulations using the final coordinates of the fully interacting lamdba window for the solvent and vacuum legs of both replicate 0 and replicate 3 for *lig_215*. Replicate 0 and replicate 3 were chosen because they have the largest difference in calculated AHFE (1.7 kcal/mol or 7.1 kJ/mol). We used gromacs v2021.2 to run these simulations. We performed an energy analysis of the Lennard-Jones Short Range (LJ-SR) and Coulomb-Short Range (Coulomb-SR), looking at how these energies changed from solvent to vacuum across charge sets. Of the overall free energy difference between charge sets, we find that 6.0 kJ/mol of the overall free energy difference is accounted for via short range interactions – in other words, most of the free energy change is due to short-ranged enthalpic interactions. Further, most of the resulting discrepancy comes from the vacuum Coulomb-SR interactions, implying that the majority of the energy difference between these two simulations is due to a conformational change of the solute in vacuum with one charge set relative to the other.

We performed a similar enthalpy analysis for an additional case, using the final coordinates of the fully interacting lamdba window for the solvent and vacuum legs of both replicate 0 and replicate 3 for lig_ejm_{43}. Replicate 2 and replicate 4 were chosen because they have the largest difference in calculated AHFE (3.8 kcal/mol or 15.9 kJ/mol). We used gromacs v2021.2 to run these simulations. As above, we performed an energy analysis of the Lennard-Jones Short Range (LJ-SR) and Coulomb-Short Range (Coulomb-SR). In this case,

Table S2: lig_ejm_{43} End-state energy analysis for Replicate 0 and 3 in solvent and vacuum

Interaction	Replicate 2 (kJ/mol)	Replicate 4 (kJ/mol)	Energy Difference (kJ/mol)
Solvent LJ-SR Solvent-Solute	-111.77	-111.62	-0.15
Solvent LJ-SR Solute-Solute	-5.60	-5.73	-0.13 Solvent Coulomb-SR Solvent-Solute
-161.47	-161.25	0.22	
Solvent Coulomb-SR Solute-Solute	-809.07	-808.67	0.40
Vacuum LJ-SR	-2.34	-2.25	-0.09
Vacuum Coulomb-SR	811.14	811.11	0.04

we find that only 0.10 kJ/mol of the free energy difference across charge sets is accounted for via enthalpy changes due to short range interactions. Indeed, enthalpy differences are overall minor, indicating that most of the difference in free energy across charge sets in this case comes from entropy differences due to the change in charge model.

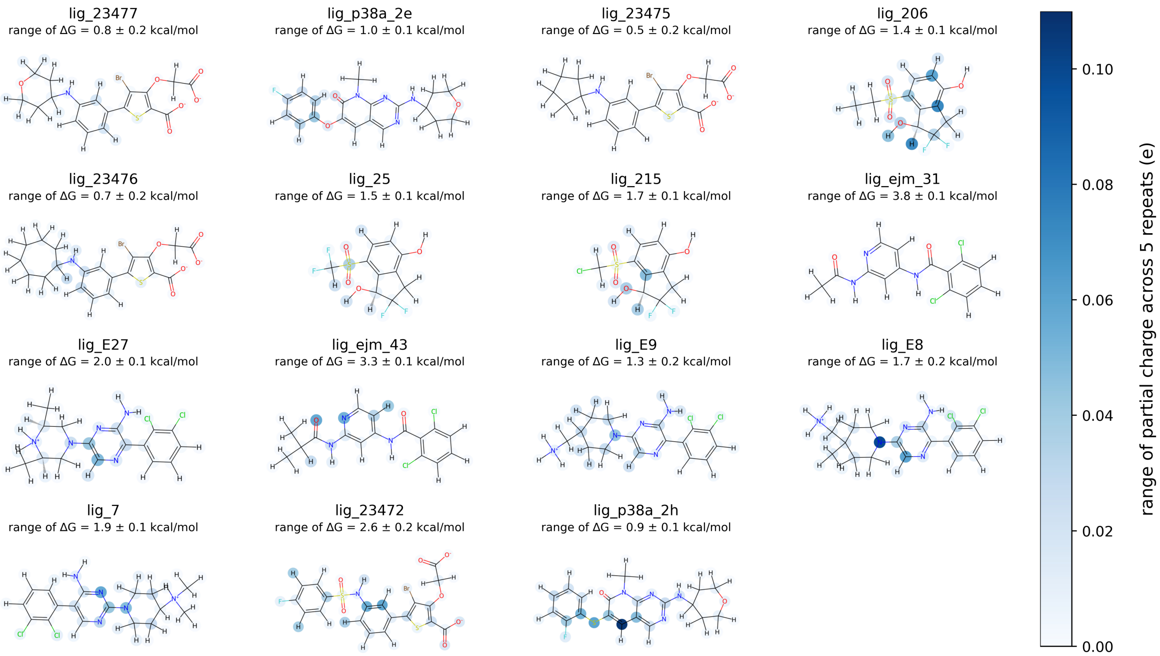

Figure S10: PLB molecules partial charge difference. The molecules in increasing order for the range of cycle closed ΔG observed. For each molecule, we generated 5 partial charge sets using AmberTools AM1-BCC charges with a random input conformer. Each atom is colored based on the legend by the range of partial charge seen at that atom.

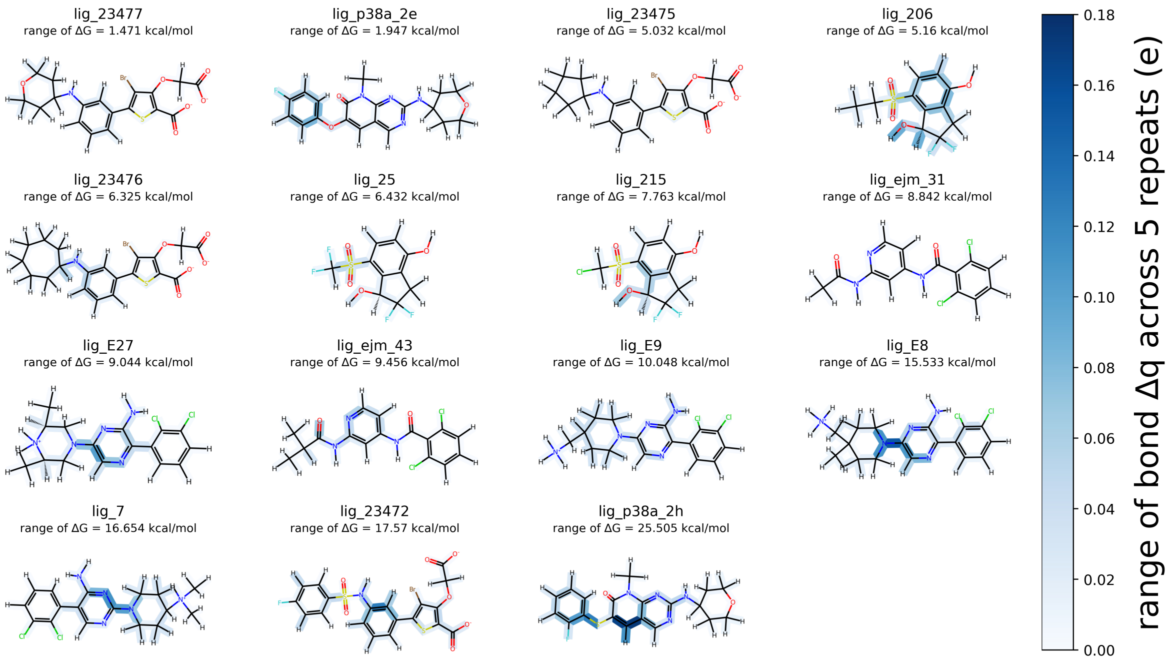

Figure S11: PLB molecules Δq_bond *difference*. The molecules in increasing order for the range of cycle closed ΔG observed. For each molecule, we generated 5 partial charge sets using AmberTools AM1-BCC charges with a random input conformer. Each bond is colored based on the legend by the range of Δq_bond seen at that atom.

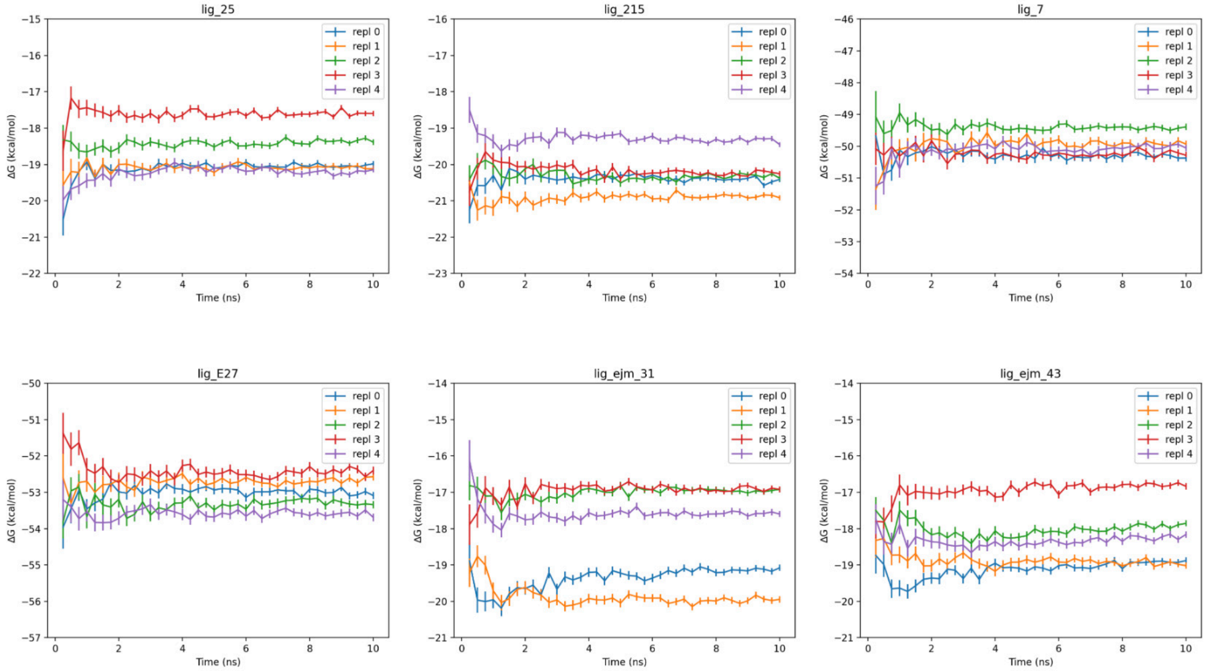

Figure S12: PLB AHFE ΔG versus time using AmberTools AM1-BCC charges from an Intel machine. We ran AHFE calculations for 6 molecules of the PLB dataset where all input conformers for partial generation were the same as the M1 mac counterparts in Figure S9 however, the partial charges were generated on an Intel machine. We find that all simulations trials seemingly converge as their ΔG versus time plots flatten out. We see a maximal range of ΔG of 4 kcal/mol.

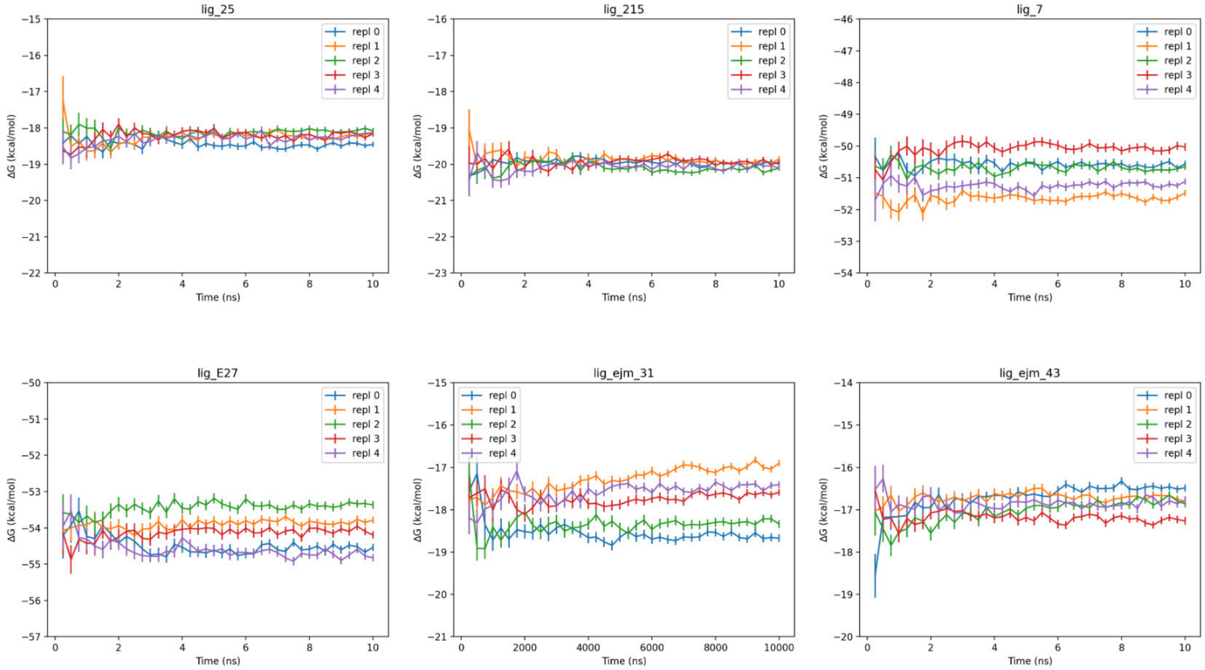

Figure S13: PLB AHFE ΔG versus time using AM1-BCC ELF10 charges. For each of the 6 molecules (the same molecules as Figure S12), we generated 5 sets of 500 random conformers and used each set of random conformers to generate a set of AM1-BCC ELF10 partial charges. We find that all simulations trials seemingly converge as their ΔG versus time plots flatten out. We see a maximal range of ΔG of 2 kcal/mol using AM1-BCC ELF10 charges

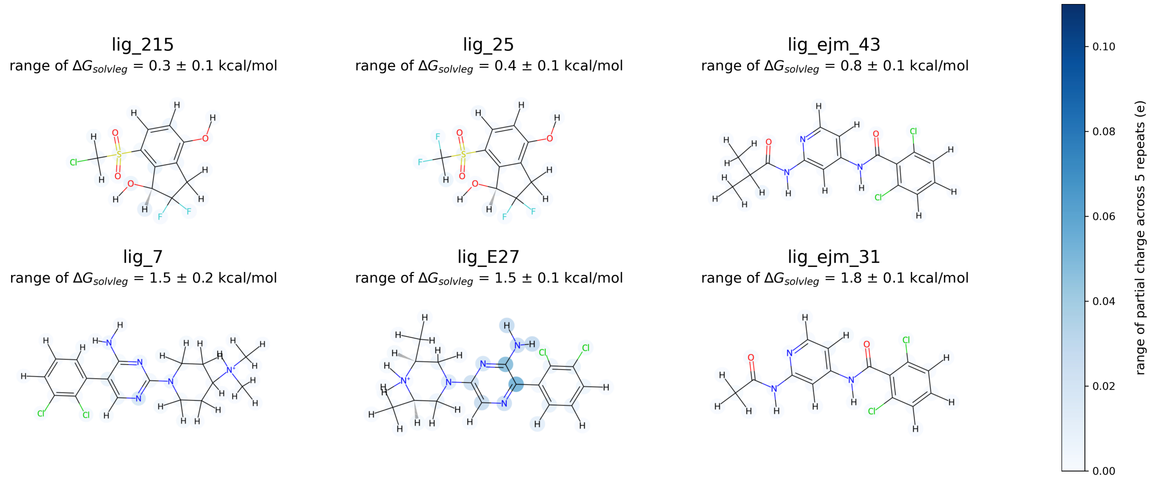

Figure S14: PLB molecules with AM1-BCC ELF10 charges Δq_bond *difference*. The molecules are in increasing order for the range of cycle closed ΔG observed. For each of the 6 molecules (the same molecules as Figure S12), we generated 5 sets of 500 random conformers and used each set of random conformers to generate a set of AM1-BCC ELF10 partial charges. Each bond is colored based on the legend by the range of Δq_bond seen at that atom.

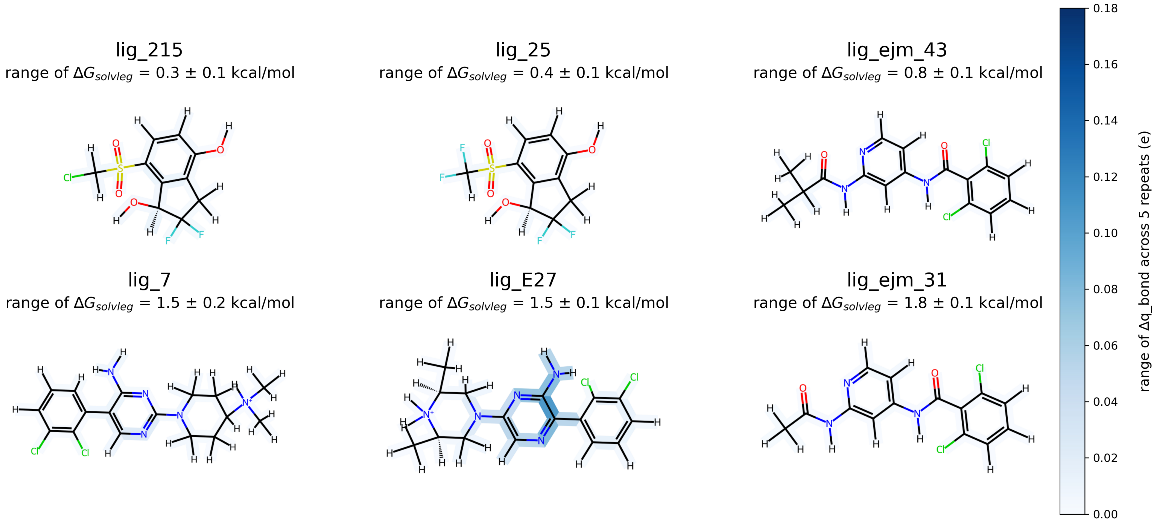

Figure S15: PLB molecules with AM1-BCC ELF10 charges Δq_bond *difference*. The molecules are in increasing order for the range of cycle closed ΔG observed. For each of the 6 molecules (the same molecules as Figure S12), we generated 5 sets of 500 random conformers and used each set of random conformers to generate a set of AM1-BCC ELF10 partial charges. Each bond is colored based on the legend by the range of Δq_bond seen at that atom.

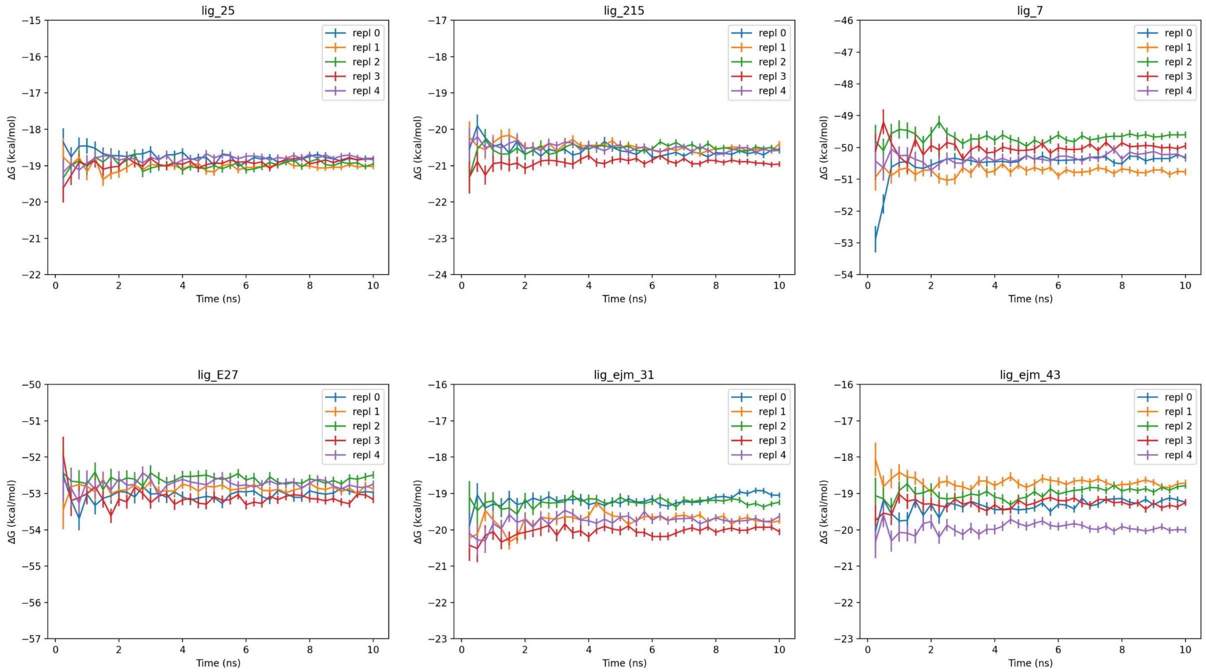

Figure S16: PLB AHFE ΔG versus time using NAGL charges. For each of the 6 molecules (the same molecules as Figure S12), we generated 5 random conformers to generate 5 partial charge sets using NAGL. We find that all simulations trials seemingly converge as their ΔG versus time plots flatten out. We see a maximal range of ΔG of 1.5 kcal/mol using AM1-BCC ELF10 charges

6.5 FreeSolv solvation free energy calculations

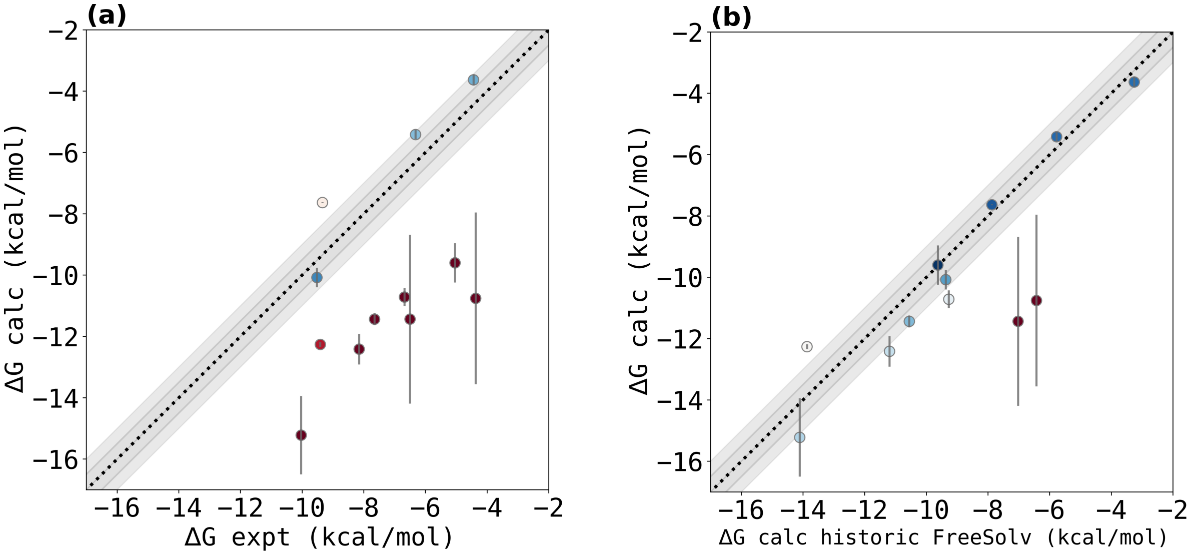

Figure S17: FreeSolv calculated ΔG compared to experimental values and historical FreeSolv calculations using AmberTools AM1-BCC charges. **(a)** Our calculated ΔG values do not agree well with experimental hydration free energy values with a root mean squared error (RMSE) from experiment of 3.8±0.6 kcal/mol **(b)** We see that 9 out of 12 of our calculations agree with previously calculated hydration free energy ΔG values from the FreeSolv database within 1.5 kcal/mol.

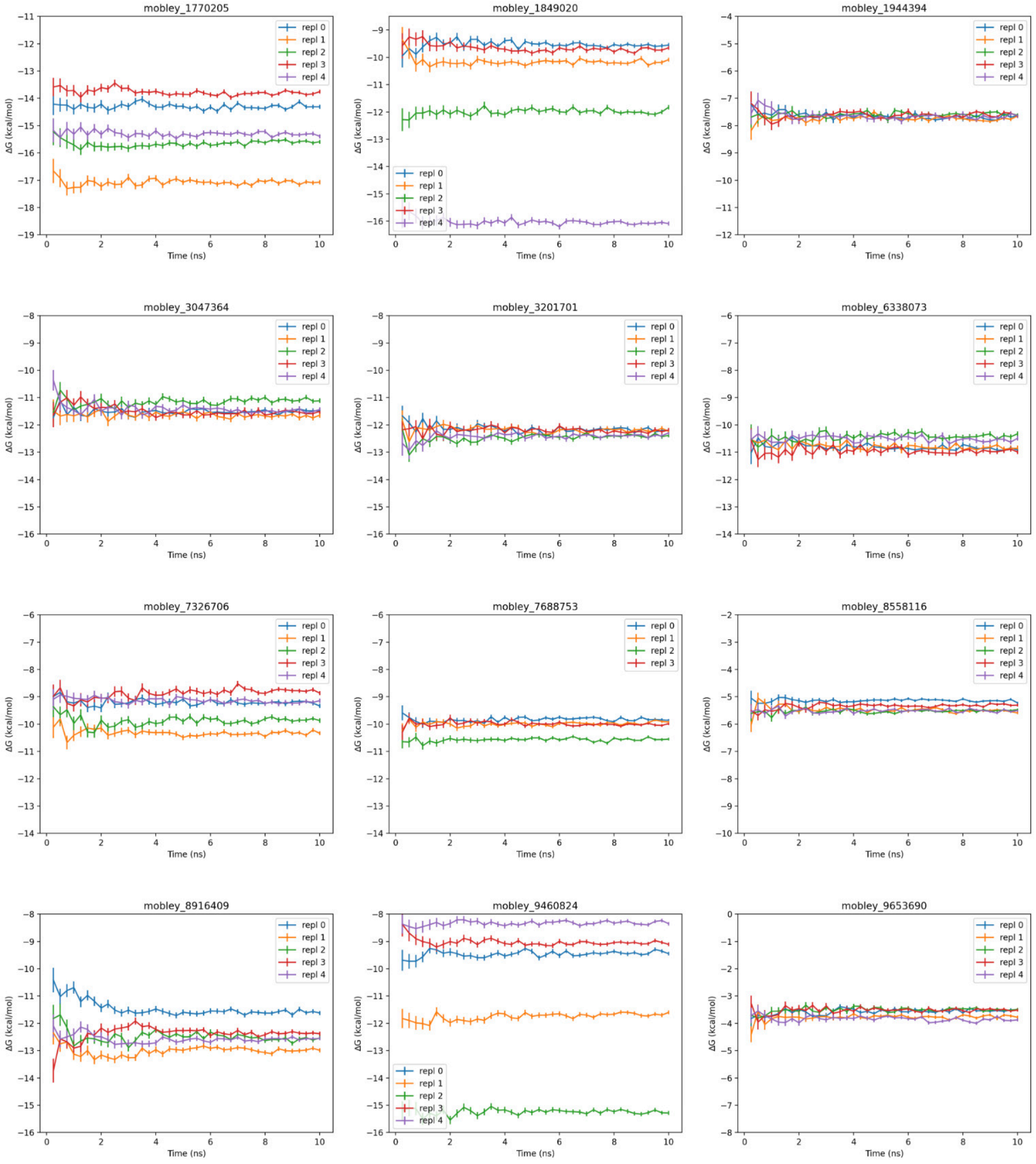

Figure S18: FreeSolv AHFE ΔG versus time using AmberTools AM1-BCC charges. We ran AHFE calculations for 12 molecules of the FreeSolv dataset. Each plot shows 5 replicates, each starting from a different partial charge set. We find that all simulations trials seemingly converge as their ΔG versus time plots flatten out.

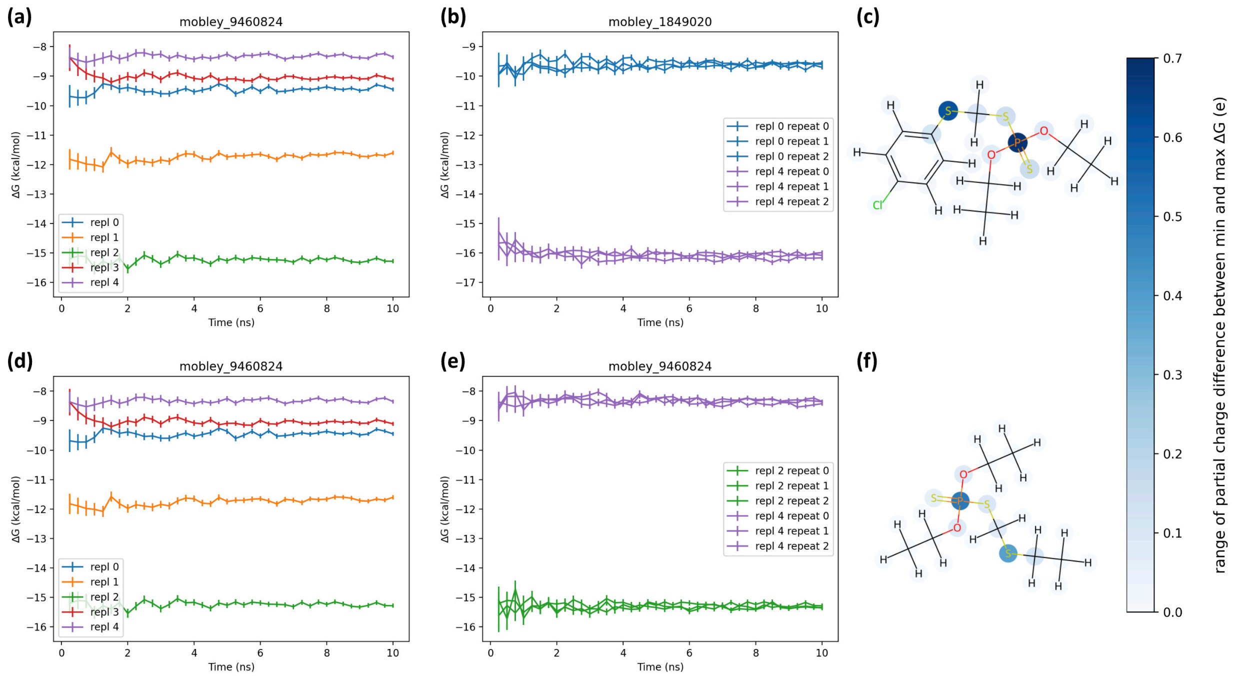

Figure S19: FreeSolv pyrophosphate extra replicates ΔG versus time using AmberTools AM1-BCC charges. Because pyrophosphates are known to behave poorly in simulation, we wanted to confirm our calculations were converged. We ran identical repeats of the most extreme replicates for 2 molecules (*mobley*_1849020 and *mobley*_9460824) from the original FreeSolv AHFE calculations. **(a)** All replicates ΔG v. Time of the FreeSolv AHFE calculations where each replicate differs only in partial charges for *mobley*_1849020. We find that replicate replicate 0 and replicate 4 are the result in the maximal and minimal calculated ΔGs. **(b)** We ran two more identical repeats with identical partial charges to replicate 0 and two more identical repeats with identical partial charges to replicate 4. We find that replicate 0 and replicate 4 seem converged across repeats. This suggests that the large partial charge difference between replicate 0 and replicate 4 the result of partial charge differences. **(c)** Here, we depict the partial charge difference at each atom between the partial charges used for replicate 0 and replicate 4. The maximum partial charge difference is 0.68 e. **(d)** All replicates ΔG v. Time of the FreeSolv AHFE calculations where each replicate differs only in partial charges for *mobley*_9460824. We find that replicate 4 and replicate 2 result in the maximal and minimal calculated ΔGs. **(e)** We ran two more identical repeats with identical partial charges to replicate 4 and two more identical repeats with identical partial charges to replicate 2. We find that replicate 0 and replicate 4 seem converged across repeats. This suggests that the large partial charge difference between replicate 4 and replicate 2 the result of partial charge differences. **(f)** Here, we depict the partial charge difference at each atom between the partial charges used for replicate 2 and replicate 4. The maximum partial charge difference is 0.49 e.

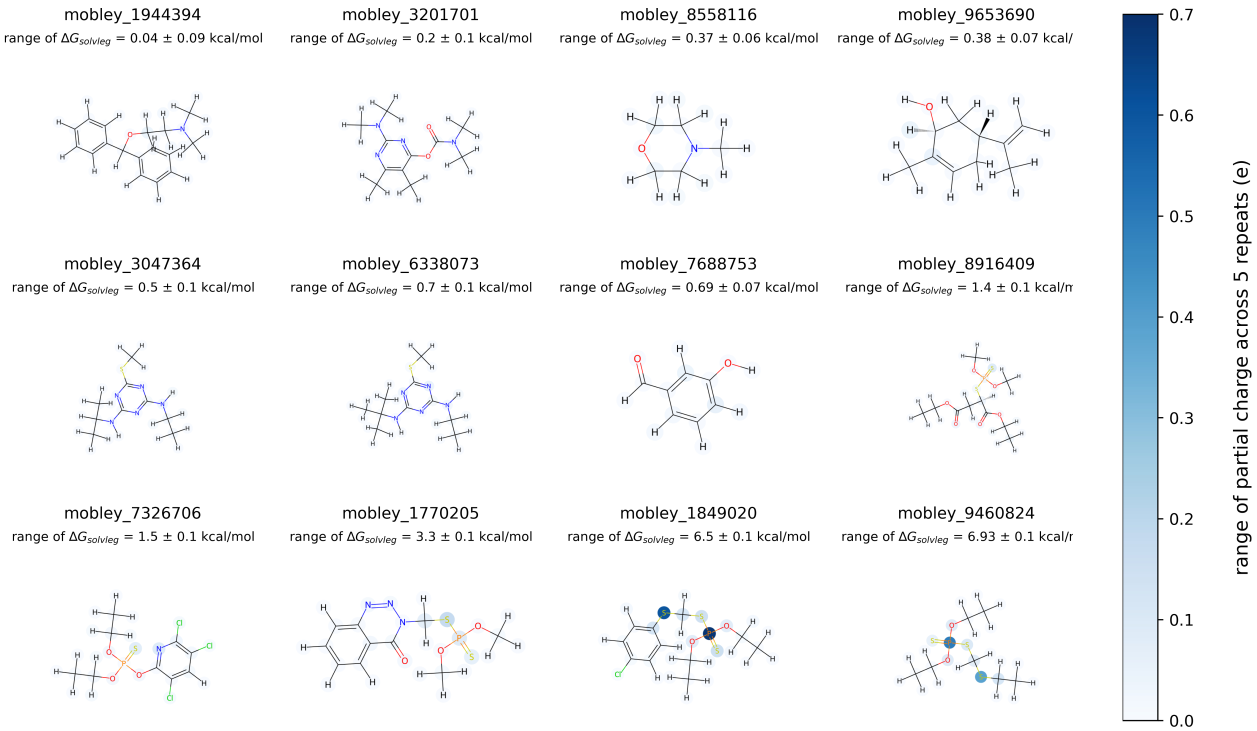

Figure S20: Freesolv molecules partial charge difference using AmberTools AM1-BCC charges. The molecules are in increasing order for the range of cycle closed ΔG observed. For each molecule, we generated 5 partial charge sets using AmberTools AM1-BCC charges with a random input conformer. Each atom is colored based on the legend by the range of partial charge seen at that atom.

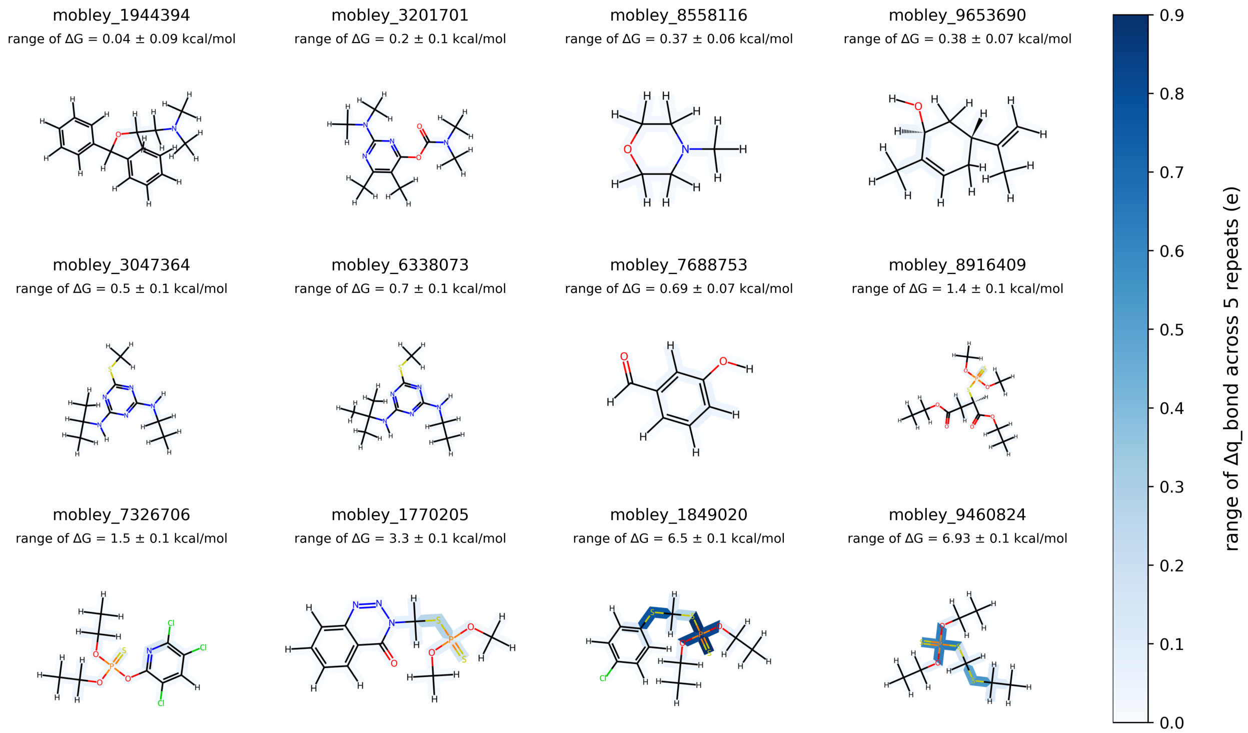

Figure S21: Freesolv molecules Δq_bond *difference* using AmberTools AM1-BCC charges. The molecules are in increasing order for the range of cycle closed ΔG observed. For each molecule, we generated 5 partial charge sets using AmberTools AM1-BCC charges with a random input conformer. Each bond is colored based on the legend by the range of Δq_bond seen at that atom.

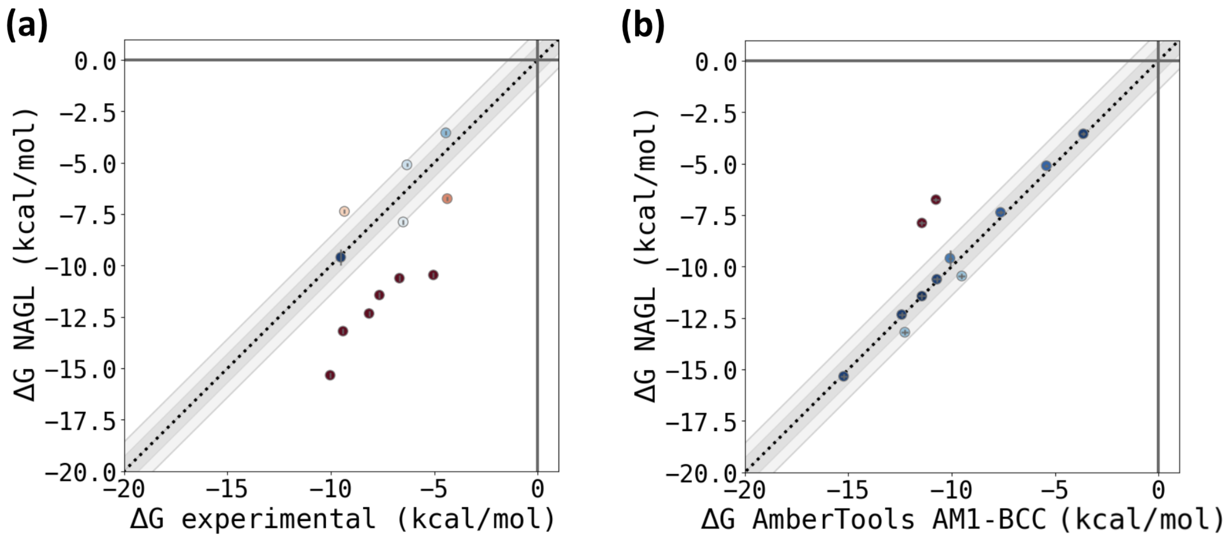

Figure S22: FreeSolv calculated ΔG compared to experimental values and FreeSolv calculations using NAGL partial charges. In general, this set of molecules is challenging for hydration free energy calculations, and agreement with experiment is not outstanding. Literature calculations gave an RMS error of 2.7 kcal/mol. **(a)** Here, our calculated ΔG values using NAGL charges do not substantially improve agreement with experimental hydration free energy values with a root mean squared error (RMSE) from experiment of 3.3 ± 0.6 kcal/mol **(b)** We see that 10 out of 12 of our NAGL calculations agree with the AHFE values calculated using AmberTools AM1-BCC charges within 1.5 kcal/mol.
